# CAR products from novel sources: a new avenue for the breakthrough in cancer immunotherapy

**DOI:** 10.3389/fimmu.2024.1378739

**Published:** 2024-04-11

**Authors:** Jiawen Huang, Qian Yang, Wen Wang, Juan Huang

**Affiliations:** Department of Hematology, Sichuan Academy of Medical Sciences and Sichuan Provincial People’s Hospital, University of Electronic Science and Technology of China, Chengdu, China

**Keywords:** chimeric antigen receptors, cellular immunotherapy, cancer treatment, tumor microenvironment, toxicity, off-the-shelf products

## Abstract

Chimeric antigen receptor (CAR) T cell therapy has transformed cancer immunotherapy. However, significant challenges limit its application beyond B cell-driven malignancies, including limited clinical efficacy, high toxicity, and complex autologous cell product manufacturing. Despite efforts to improve CAR T cell therapy outcomes, there is a growing interest in utilizing alternative immune cells to develop CAR cells. These immune cells offer several advantages, such as major histocompatibility complex (MHC)-independent function, tumor microenvironment (TME) modulation, and increased tissue infiltration capabilities. Currently, CAR products from various T cell subtypes, innate immune cells, hematopoietic progenitor cells, and even exosomes are being explored. These CAR products often show enhanced antitumor efficacy, diminished toxicity, and superior tumor penetration. With these benefits in mind, numerous clinical trials are underway to access the potential of these innovative CAR cells. This review aims to thoroughly examine the advantages, challenges, and existing insights on these new CAR products in cancer treatment.

## 1 Introduction

Cancer represents a significant global public health challenge and remains one of the primary causes of mortality worldwide. According to the International Agency for Research on Cancer, there are approximately 19.3 million new cancer diagnoses and nearly 10.0 million cancer-related deaths each year (1). The development of cellular immunotherapy has profoundly changed cancer treatments. Inspired by allogeneic hematopoietic stem cell transplantation (HSCT), researchers have harnessed the immune system to target and eliminate cancer cells. This is achieved through the use of chimeric antigen receptors (CARs), synthetic receptors that redirect T cell specifically against cells with certain antigens. Thus, CAR T cell therapy has become a major breakthrough in immunotherapy (1).

Despite its notable successes, CAR T cells’ exceptional efficacy is mainly limited to B cell-driven hematological malignancies. Expanding its applicability to a wider range of cancers faces numerous obstacles (2). For instance, tumor cells frequently develop complex mechanisms to evade eradication, such as loss-of-antigen. It is reported that CD19-negative relapse accounts for around 50% of relapse after CD19-CAR T cell therapy in patients with B cell-driven malignancy (3). The tumor microenvironment (TME) frequently exhibits immunosuppressive characteristics. These include the presence of immunosuppressive cells and upregulated inhibitory immune checkpoints, which can compromise the effectiveness of CAR T cells *in vivo*. Continuous and intense stimulation may make T cells prone to exhaustion, raising concerns about the long-term efficacy and persistence of CAR T cells in tumors. Some clinical trials even reported that their mesothelin-CAR T cells can only persist in patient’s body for 28 days, resulting undesirable outcomes (4). In solid tumors, CAR T cells often exhibit suboptimal tumor infiltration. Besides, producing autologous cell products is labor-intensive and time-consuming. The starting material of the autologous products varies in each therapy, adding to the complexity. Importantly, CAR T cell therapy is associated with severe adverse effects. Life-threatening complications, such as cytokine release syndrome (CRS) and immune effector cell-associated neurotoxicity syndrome (ICANS), often lead to treatment failures (2).

The immune system is a complex defense network, encompassing a wide range of immune cells. They come equipped with a variety of capacities, and these inherent properties may help overcome the challenges of conventional CAR T cell therapies. As a result, there is increasing interest in introducing CAR constructs into various immune cells. This review will focus on the latest and most promising advancements in new CAR cell therapies.

## 2 CAR cells generated from various T cell subtypes

The polyclonal nature of conventional CAR T cells’ endogenous TCRs introduces a heightened risk of off-target effects (5). Additionally, their antitumor activity is largely dependent on CAR receptors, which can be undermined by antigen loss, CAR downregulation, or the immunosuppressive effects of the tumor microenvironment (TME) (6). These factors raise significant concerns regarding the *in vivo* efficacy and potential toxicities of CAR T cell therapies. However, leveraging specific T cell subtypes to create CAR constructs could offer a solution. These new T cell-derived CAR cells might possess intrinsic, CAR-independent cytotoxic capabilities.

### 2.1 CAR NKT cells

#### 2.1.1 Properties and advantages

**Figure 1:**
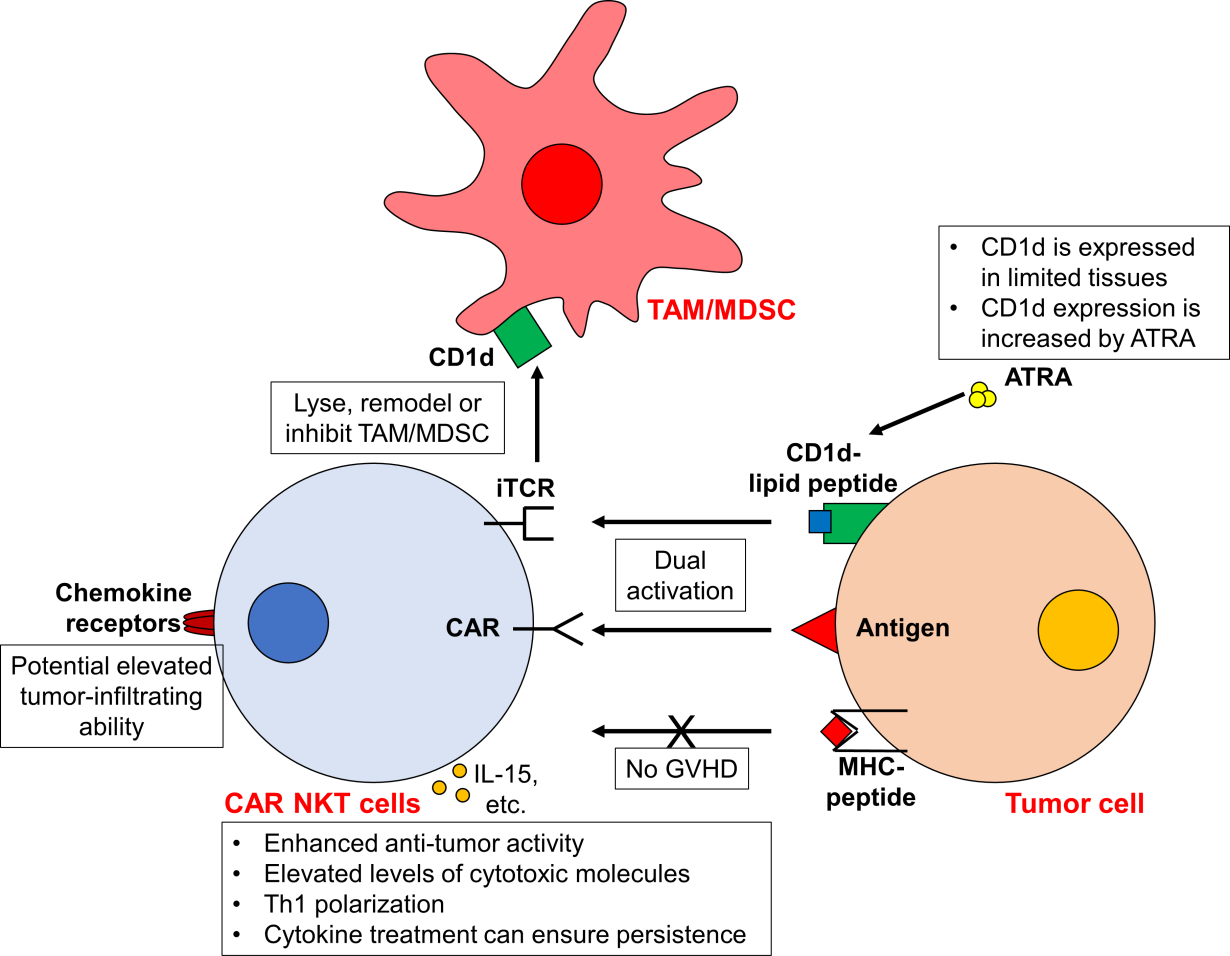
CAR NKT cells exhibit enhanced antitumor activity against tumor cells. Due to the unique iTCR-CD1d interaction, CAR NKT cells display both CAR-dependent and CAR-independent (i.e. iTCR-dependent) cytotoxicity, leading to enhanced antitumor activity. The expression of CD1d in tumor cells can be increased using ATRA, further boosting the iTCR-dependent activity. Furthermore, CAR NKT cells can target TAMs/MDSCs in a CD1d-dependent manner. Moreover, as the activity of iTCR is MHC-independent, the risk of GVHD is low. Upon activation, these CAR NKT cells are Th1 polarized and express higher cytotoxic molecules. The administration of cytokines (such as IL-15) may prolong the *in vivo* persistence of CAR NKT cells. In addition, the elevated expression of chemokine receptors on NKT cells may confer the ability of CAR NKT cells to infiltrate into tumors. However, it should be noted that CD1d is only expressed in certain tissues and cell types. While the toxicity from CAR NKT cells is limited, concerns arise that CAR NKT cells is probably only applicable to CD1d^+^ tumors.

Natural killer T (NKT) cells play a crucial role in innate tumor surveillance and exhibit significant antitumor activity (7–9). Type I or invariant NKT (iNKT) cells are the major subset of NKT cells with an identical or invariant T cell receptor (iTCR). This receptor uniquely recognizes lipid antigens presented by the non-polymorphic molecule CD1d, instead of traditional MHC molecules (10–12). This distinction provides several benefits for developing CAR cells from NKT cells. Specifically, the iTCR-CD1d interaction empowers CAR NKT cells to retain robust TCR signaling, enabling antitumor responses that extend beyond CAR activity (13). Additionally, the inherent CAR-independent cytotoxicity of iNKT-derived CAR NKT cells is naturally limited by CD1d expression (14, 15). This restriction helps confine potential toxicities associated with iNKT cell activity primarily to CD1d-positive tissues (13). Moreover, CAR NKT cells may alter the immunosuppressive TME in a CD1d-dependent manner. Despite limited CD1d expression in most human tumors, tumor-associated macrophages (TAMs) in various cancers do express CD1d, allowing iNKT cells to either lyse (16) or remodel (17, 18) these TAMs to enhance antitumor responses. Furthermore, in influenza A infections (19) or breast cancer (20), iNKT cells can inhibit myeloid-derived suppressor cells (MDSC) via CD1d interactions, mitigating immunosuppression (**Figure 1**).

In addition to the CD1d-related advantages, iNKT cells also stand out for their enhanced ability to migrate towards tumors. This is due to their high levels of chemokine receptors such as CCR1, CCR2, CCR4, CCR5, CCR6, CXCR3 and CXCR4, compared to conventional T cells (21) (**Figure 1**). For example, the expression of CCR4 facilitates the skin homing of T cells, which may direct CAR iNKT cells towards skin-associated malignancies (22). The high expression of CXCR4 on iNKT cells can promote bone marrow migration as well (23). Moreover, research has documented a notable presence of tumor-infiltrating human NKT cells in a murine lymphoid tumor model (24). Furthermore, increased NKT cell infiltration within tumors is correlated with improved clinical outcomes (25–30). However, a high degree of heterogeneity exists within iNKT cells. While CXCR3 and CXCR4 are expressed in over 90% of iNKT cells, only 30% of iNKT cells express CCR4 (which are mostly CD4^+^ iNKT cells). The expression of other chemokine receptors varies as well (21). Therefore, the tumor infiltration ability of each CAR NKT cell should be carefully assessed. The function of different iNKT cell subtypes is also different. For example, while CD4^+^ iNKT cells secrete both Th1 and Th2 cytokines, CD4^-^CD8^-^ and CD8^+^ iNKT cells only generate Th1 cytokines (21). Whether this heterogeneity affect the activity of CAR iNKT cells awaits further investigation. It may be necessary to isolate subtypes of iNKT cells for CAR transduction.

Another promising benefit of CAR NKT cells is the potential allogeneic applications (**Figure 1**). This is because NKT cell activity does not depend on MHC molecules. Researches have shown that donor-derived NKT cells can mitigate acute graft-versus-host disease (aGVHD) in animal models and clinical trials after HSCT (31–36). For instance, CD4^-^ NKT cells can suppress T cell proliferation and IFN-γ secretion through direct contact (34). Similarly, CD4^+^ NKT cells not only suppress effector T cell proliferation but also retain their graft-versus-tumor effects (33) and facilitate regulatory T cell expansion (31). Therefore, the risk of allogeneic CAR NKT cells inducing severe GVHD appears minimal. However, whether this Treg-like function affects the antitumor activity of CAR NKT cells and endogenous immune cells requires further investigation.

Peripheral blood mononuclear cells (PBMCs) are commonly used as the starting materials for generating CAR NKT cells. However, there is still no consensus on the specific procedure for processing NKT cells. For example, NKT cells can be isolated using TCRVα24^+^TCRVβ11^+^ marker (0.13% of PBMCs) (13), Vα24-Jβ18^+^ marker (0.225% of PBMCs) (37), anti-iNKT microbeads (TCR α-chain Vα24-Jα18, percentage not reported) (38), or CD3^+^CD56^+^ NKT cell isolation kit (2% of PBMCs) (39). Once isolated, NKT cells can be transduced directly or after initial expansion. Such expansion is achieved by using anti-CD3/CD28-mediated stimulation and/or iNKT cell agonist alpha-galactosylceramide (αGalCer)-pulsed cells, in the presence of IL-15 or IL-2 (13, 38, 39). Rotolo et al. conducted a study comparing different protocols and suggested that TCRVα24^+^TCRVβ11^+^ selection, followed by CD3/CD28-activation in the presence of autologous antigen-presenting cells (APCs) and IL-15, can effectively generate viable CAR NKT cells (13). However, further investigations are still needed.

#### 2.1.2 Current study

Exploiting the unique properties of NKT cells, numerous preclinical studies have focused on creating diverse CAR NKT cells to target various tumors. For instance, CD19 and CD1d are commonly expressed in various B cell malignancies (40). Building on this, Rotolo et al. engineered CD19-CAR NKT cells to specifically target CD19^+^ B cell lymphomas (13). These engineered cells achieved enhanced anti-lymphoma effects *in vitro* and *in vivo* by activating both the iTCR-CD1d and CAR-CD19 signaling pathways (13). Compared to conventional CD19-CAR T cells, CD19-CAR NKT cells manifested elevated levels of cytotoxic molecules such as IFNγ, perforin, and granzymes and displayed a pronounced Th1 polarization. Furthermore, the study also found that increasing *CD1D* expression in human B cells with the RARα ligand all-trans retinoic acid (ATRA) can increase the cytotoxicity of CD19-CAR NKT cells (13). Similarly, GD2-targeted CAR NKT cells designed for neuroblastoma exhibited inherent iTCR-dependent activity, including the ability to target TAMs, and demonstrated Th1 polarization (38). Additionally, Simon et al. highlighted that chondroitin sulfate proteoglycan 4 (CSPG4)-specific CAR NKT cells, designed for melanoma, maintained the ability to eliminate target cells through their endogenous TCRs, a mechanism independent from CAR-induced activity. These cells showed a comparable, if not superior, cytotoxicity relative to traditional CAR T cells (39). Likewise, CD38- and BCMA-CAR NKT cells exhibited dual CAR-dependent and iTCR-dependent activity against multiple myeloma (MM) cells (41). These examples underline the potential of CAR NKT cells for potentiated antitumor activity with reduced off-tumor effects (41).

The enhanced expression of chemokine receptors on CAR NKT cells potentially increases their tumor infiltration ability. Specifically, both CD19-CAR NKT cells and GD2-CAR NKT cells have demonstrated superior tumor infiltration ability in solid tumors when compared to the conventional CAR T cells (13, 38, 42). Moreover, the increased expression of ITGA4 and ITGB1 in CAR NKT cells facilitates the passage through the blood-brain barrier and supports the eradication of brain tumors (13).

For allogeneic or off-the-shelf applications, the infusion of humanized CAR NKT cells does not lead to clinically notable xenograft GVHD in mouse xenograft tumor models (13, 38). Such complications are routinely observed following the administration of conventional CAR T cells (43). This observation suggests the potential for allogeneic CAR NKT cells to similarly avoid inducing acute GVHD in human. Furthermore, the inherent immunomodulatory properties of CAR NKT cells may help to reduce adverse effect elicited by endogenous immune cells. However, further studies are essential to substantiate this hypothesis (**Figure 1**).

#### 2.1.3 Challenges & solutions

While CAR NKT cells present a promising therapeutic intervention, two primary challenges persist. Firstly, the function of CAR NKT cells is somewhat dependent on CD1d, which is limited to specific cell types (14, 15). While this reliance restricts off-target toxicity, it also confines their effectiveness largely to CD1d^+^ tumors. Furthermore, loss-of-antigen relapse is a common cause of failure in CAR cell therapy (44). During CAR NKT cell treatment, tumor cells might downregulate their CD1d expression. However, this may not represent an insurmountable barrier for CAR NKT cells. Typically, B cell chronic lymphocytic leukemia (CLL) cells have low or absent CD1d expression (29, 40). Yet, they can still be targeted by CD19-CAR NKT cells via both CAR and CD1d-dependent mechanism (13). The application of ATRA to modulate CD1d expression highlights a method to enhance both the efficacy and safety of CAR NKT cell treatments as well (13). Complementing this observation, Heczey et al. showed that GD2-CAR NKT cells could effectively target GD2^+^CD1d^-^ neuroblast cells (38). This suggests that CAR NKT cells can operate purely through CAR-induced signaling, akin to traditional CAR T cells. Nevertheless, whether the effectiveness of such CAR-mediated cytotoxicity is comparable with that of conventional CAR T cells requires further exploration.

Another primary challenge is the transient persistence of CAR NKT cells, often requiring repeat doses for sustain tumor control (38). Conventional CAR T cells benefit from design improvements, such as the tailored design of costimulatory domains, and the cytokines treatment like IL-15 (44). Similar efforts have been made for CAR NKT cells to extend their durability (38, 42) (**Figure 1**). Furthermore, CD62L^+^ NKT cells exhibit markedly superior proliferative capabilities compared to the CD62L^-^ NKT cells. Leveraging CD62L^+^ cells for CAR NKT cell production results in enhanced persistence and antitumor effects, as evidenced in murine lymphoma and neuroblastoma models (45). Liu et al. further demonstrated that these memory-like CD62L^+^ NKT cells can be induced upon cytokine administration (46). By incorporating IL-21 into B7H3-CAR NKT cells, they increased the frequency of CD62L^+^ subsets in these CAR NKT cells. In mouse renal cancer xerograph models, such IL-21-armored B7H3-CAR NKT cells showed reduced exhaustion, significantly prolonged proliferation and extended persistence without obvious adverse effects (46). These insights suggest new avenues for optimizing the long-term efficacy of CAR NKT cells (**Table 1**).

**Table 1:** Advantages and limitations of novel CAR products.

CAR products	Advantages	Limitations	Examples of possible tumor-killing types
CAR NKT cells	1. enhanced antitumor activity due to CAR-independent function 2. improved safety 3. ability to remodel the immune-suppressive TME 4. increased tumor infiltration 5. potential application of allogeneic products	1. the activity may be CD1d-restricted 2. reduced persistence	B cell malignancies, melanoma, cutaneous and subcutaneous malignancies, neuroblastoma, glioblastoma
CAR MAIT cells	1. potent antitumor activity due to CAR-independent function 2. improved safety 3. ability to remodel the immune-suppressive TME 4. increased tumor infiltration (not proven in CAR cells yet) 5. potential application of allogeneic products 6. presence of novel subset with effector memory phenotype (not proven in CAR cells yet)	1. lack of *in vivo* study 2. increased on-target, off-tumor activity in some studies	B cell malignancies, breast cancers, ovarian cancer
CAR γδ T cells	1. potent antitumor activity due to CAR-independent function 2. potential application of allogeneic products 3. increased tumor infiltration (for CAR Vδ1 T cells, further study required) 4. ability to boost endogenous immunity 5. ease of *ex vivo* and *in vivo* expansion via amino bisphosphonates administration	1. the activity is not superior over conventional CAR T cells 2. the intrinsic antitumor activity may be limited to certain tumor types. 3. reduced persistence 4. CAR Vδ2 T cells are prone to exhaustion 5. poor *in vivo* expansion due to weak alloreactivity	B cell malignancies, neuroblastoma, AML, T cell malignancies, glioblastoma, hepatocellular carcinoma, ovarian cancer
CAR CIK cells	1. ease of *ex vivo* expansion 2. potent antitumor activity due to CAR-independent function 3. improved safety 4. potential application of allogeneic products 5. increased tumor infiltration (not proven in CAR cells yet)	1. weaker *in vivo* activity than conventional CAR T cells 2. poor persistence due to terminal differentiation 3. tricky CAR construct design	Rhabdomyosarcoma, nasopharyngeal carcinoma, melanoma, soft tissue sarcoma, cutaneous and subcutaneous malignancies, B cell malignancies
CAR NK cells	1. potent antitumor activity due to CAR-independent function 2. improved safety 3. potential application of allogeneic products	1. poor *in vivo* persistence	Various hematological malignancies and solid tumors (including renal cell carcinoma, ovarian cancer, breast cancer, AML, etc.)
CAR macrophages	1. enhanced antitumor activity due to CAR-independent function 2. ability to remodel the immune-suppressive TME 3. improved safety 4. increased tumor infiltration 5. potential application of allogeneic products 6. synergistic effect with CAR T cells	1. alternative sources of cells for CAR transduction (with safety issues) 2. poor *in vitro* and *in vivo* proliferation 3. complex CAR construct design 4. uncertainty of the resistance to the M2-inducing TME 5. challenging genetic manipulation	Breast cancer, gastric cancer, glioblastoma, neuroblastoma, B cell malignancies, pancreatic cancer, ovarian cancer
CAR neutrophils	1. enhanced antitumor activity due to CAR-independent function 2. ability to remodel the immune-suppressive TME 3. improved safety 4. increased tumor infiltration 5. potential application of allogeneic products	1. alternative sources of cells for CAR transduction (with safety issues) 2. poor persistence	Only examined for glioblastoma, prostate cancers
CAR HSPCs	1. prolonged persistence 2. ability to generate almost all types of CAR immune cells	1. impaired T cell differentiation 2. potential biased HSPC development	Only examined for B cell malignancies
Exosomes from CAR cells	1. potent antitumor activity 2. increased tumor infiltration 3. potential application of allogeneic products 4. resistance to the immune-suppressive TME 5. improved safety	1. unknown mechanism of cytotoxicity 2. difficulty of determining the amount needed 3. undetermined long-term effect	B cell malignancies, breast cancer, lung adenocarcinoma

#### 2.1.4 Ongoing clinical trials

Presently, two phase I clinical trials have published interim results: one evaluates autologous GD2-CAR NKT cells with IL-15 in children with relapsed or resistant neuroblastoma (NCT03294954), while the other focuses on allogeneic CD19-CAR NKT cells with IL-15 targeting relapse and refractory B cell malignancies (NCT03774654). Preliminary analysis of the former trial (in 2020) highlighted promising aspects such as *in vivo* expansion, tumor infiltration, and lack of dose-limiting toxicity associated with GD2-CAR NKT cells (47). However, merely one out of the 11 participants showed an objective response (47). In their latest interim reports (in 2023), the number of patients enrolled has increased to 12, and the objective response rate reached 25% (3/12, including 2 partial responses and 1 complete response) (48). It is noteworthy that the frequency of memory-like CD62L^+^ subsets is positively correlated with the *in vivo* expansion and therapeutic outcomes, further suggesting that the persistence is one of the limiting factors for CAR NKT cell therapies. The team also identified that BTG anti-proliferation factor 1 (BTG1) as a key driver for hyporesponsiveness in CAR NKT cells (48). Meanwhile, allogeneic CD19-CAR NKT cells demonstrated both safety and *in vivo* expansion, yielding a complete response rate of 40% and a partial response rate of 40% (49). Another two clinical studies are actively assessing the efficacy of iNKT cells that co-express both CD19-CAR and IL-15, specifically targeting relapsed/refractory (r/r) or high-risk B cell tumors (NCT04814004 and NCT05487651) (**Table 2**).

**Table 2:** Clinical trials of new CAR cells.

CAR cells	CAR Target	Target cancers	Cell source and modification	Trial number	Phase*	Cell number transferred
CAR NKT cells	GD2	Relapsed or resistant neuroblastoma	Autologous; with IL-15	NCT03294954	Phase I; with interim results	3×10^6^/m^2^ of body surface
	CD19	Relapse and refractory B cell malignancies	Allogeneic; with IL-15	NCT03774654	Phase I; with interim results	10^7^ or 3×10^7^/m^2^ of body surface
	CD19	Relapsed/refractory/high-risk B cell tumors	Allogeneic; with IL-15	NCT04814004	Phase I	Not disclosed
	CD19	Refractory/relapsed B cell NHL or leukemia (ALL or CLL)	Allogeneic; with IL-15	NCT05487651	Phase I	10^7^, 3×10^7^ or 10^8^/m^2^ of body surface
CAR γδ T cells	CD20	Relapsed/refractory advanced B cell lymphoma	Allogeneic	NCT04735471	Phase I; with interim results	3 + 3 dose-escalation scheme (three dose levels: 3×10^7^, 10^8^ or 3×10^8^ cells)
	NKGD2DL	Relapsed or refractory solid tumors	Haploidentical or allogeneic	NCT04107142	Phase I	3 + 3 dose-escalation scheme (three dose levels from 3×10^8^ to 3×10^9^ cells);
	CD19	Refractory/relapsed B cell NHL or leukemia (ALL or CLL)	Allogeneic	NCT02656147	Phase I	Not disclosed
	CD7	Relapsed or refractory CD7^+^ T cell malignancies	Not disclosed	NCT04702841	Phase I	0.2 to 5×10^6^/kg
CAR CIK cells	CD19	Relapsed or refractory ALL post-HSCT	Allogeneic	NCT03389035	Phase I/II; with interim results	15×10^6^/kg (with three trial doses for the first 9 patients: 1×10^6^, 3×10^6^ or 7.5×10^6^/kg)
	CD19	Relapsed/refractory B-cell NHL or CLL	Haploidentical	NCT05869279	Phase I/II	Not disclosed
CAR macrophages	HER2	Advanced HER2-overexpressing solid tumors	Differentiation of monocytes sourced from mobilized apheresis products	NCT04660929	Phase I; with interim results	Single dose (5×10^9^) or three doses (5×10^8^ on Day 1, 1.5×10^9^ on Day 3 and 3×10^9^ on Day 5)

*All trials are ongoing.

### 2.2 CAR MAIT cells

#### 2.2.1 Properties and advantages

**Figure 2:**
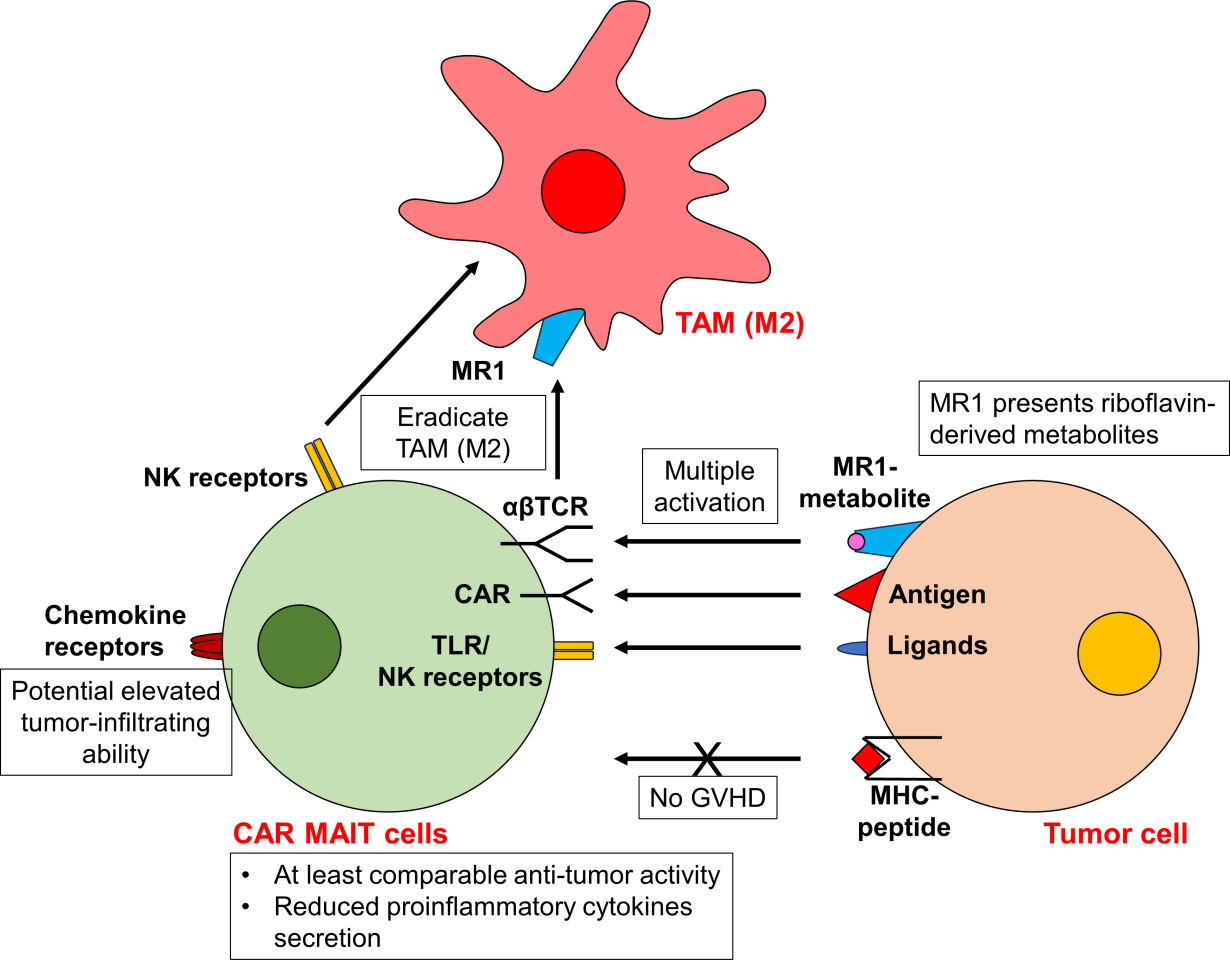
CAR MAIT cells display potent antitumor activity. Similar to CAR NKT cells, CAR MAIT cells can be activated via multiple mechanisms, in addition to conventional CAR-dependent signal. The αβTCR of CAR MAIT cells recognizes riboflavin-derived metabolites presented by the MHC class I-like protein MR1. CAR MAIT cells also express innate cell receptor TLRs and NK cell-activating receptors, facilitating their cytotoxicity even in the absence of MR1. As the activity of αβTCR is MHC-independent, it is unlikely for CAR MAIT cells to induce GVHD. Activated CAR MAIT cells exhibit at least comparable antitumor activity to conventional CAR T cells. They also release lower proinflammatory cytokines, suggesting an improved safety profile. In addition, CAR MAIT cells may be able to eliminate M2 macrophages via their αβTCR and NK cell-activating receptors. The highly expressed chemokine receptors on CAR MAIT cells also allow cells to infiltrate into peripheral tissues and tumors.

Similar to iNKT cells, mucosal-associated invariant T (MAIT) cells express a semi-invariant αβTCR with restricted repertoire. Instead of engaging with MHC molecules, the αβTCR of MAIT cells recognizes metabolite antigens presented by the MHC class I-like protein (MR1). These antigens are riboflavin-derived metabolites rather than peptides (50). MR1 is predominantly expressed in antigen-presenting cells, such as macrophages, dendritic cells, and monocytes, as well as epithelial cells. Upon activation, MAIT cells exhibit potent cytotoxic activity, directly killing target cells via the perforin/granzyme B pathways and the secretion of various proinflammatory cytokines (50). Beyond their MR1-restricted αβTCR, MAIT cells also express innate cell receptors such as Toll-like receptors (TLRs) and NK cell-activating receptors. This equips them with enhanced cytotoxic capabilities, even in the absence of MR1 (37, 50, 51). Thus, similar to CAR NKT cells, CAR MAIT cells may be activated through multiple mechanisms, culminating in augmented efficacy. Furthermore, MAIT cells proficiently target and eradicate MR1^+^ M2 polarized macrophages during co-culture, operating via a TCR-dependent and TCR-independent mechanism. This suggests a promising avenue for MAIT cells to remodel the immunosuppressive TME (37) (**Figure 2**).

Apart from their CAR-independent cytotoxicity, MAIT cells offer another significant advantage: their high expression of chemokine receptors such as CXCR6 and CCR9 (52, 53). This trait empowers them to efficiently migrate into peripheral tissues and tumors. Indeed, substantial infiltration of MAIT cells has been observed within the TME (54–56). Moreover, due to the non-polymorphic nature of MR1, MAIT cells are devoid of alloreactivity, similar to the behavior observed in NKT cells (57). Clinical data further supports this, showing an association between increased MAIT cells in grafts and reduced incidence of GVHD following HSCT (58–61). This positions MAIT cells as attractive candidates for allogeneic CAR cell therapies, with a lower risk of GVHD (**Figure 2**).

These advantages are related to MR1-dependent activation, parallel those observed with CAR NKT cells, which rely on CD1d. Furthermore, a specific MAIT cell subset characterized as CD45RA^−^CD45RO^+^CD62L^low^CD161^+^ displays an effector memory phenotype, predisposing them for rapid expansion upon activation (53, 62). This feature suggests that both MAIT cells and their engineered CAR counterparts may achieve prolonged persistence *in vivo*.

Similar to CAR NKT cells, CAR MAIT cells are also derived from PBMCs. MAIT cells are isolated based on the positive expression of TCR Vα7.2 (2.85% of PBMCs) (37) or Vα7.2^+^CD161^+^CD8^+^ expression (17.7% of CD8^+^ cells in PBMCs, although the authors used Vα7.2^+^ CD4^-^ expression as well) (63). After isolation, MAIT cells are activated and expanded in the presence of riboflavin-derived metabolites, such as 5-(2-oxopropylideneamino)-6-d-ribitylaminouracil (5-OP-RU) or 5-amino-6-d-ribitylaminouracil (5-ARU), as well as IL-2, IL-7 and/or IL-15 (37, 63).

#### 2.2.2 Current study

To date, CD19-, Her2- and mesothelin-specific CAR MAIT cells have been generated (37, 63, 64). In a comparative study, Dogan et al. observed that CD19-CAR MAIT cells displayed only marginally enhanced *in vitro* cytotoxicity against CD19^+^ cell lines (T2 and Nalm6) than conventional CAR T cells at specific effector: tumor (E:T) ratios (63). However, these CD19-CAR MAIT cells demonstrated pronounced cytotoxicity against primary B cells across all E:T ratios (63), raising concerns about potential on-target toxicity. In another aspect of their research, Dogan et al. developed Her2-CAR MAIT cells against breast cancers, and discovered that these cells displayed greater cytotoxicity than CAR T cells against MDA-231 cells at various E:T ratios (63). Notably, despite their comparable cytotoxic abilities, activated CAR MAIT cells secreted significantly lower levels of proinflammatory cytokines than CAR T cells (63). This suggests CAR MAIT cells might have an improved safety profile. Furthermore, CD19-CAR MAIT cells did not trigger GVHD in xenograft models (64), underscoring their potential allogeneic application. However, a comprehensive safety assessment is still needed. (**Figure 2**).

Beyond CD19- and Her2-CAR MAIT cells, Li et al. generated mesothelin-specific CAR MAIT cells (37). These CAR MAIT cells proficiently targeted and eradicated the mesothelin-overexpressing ovarian cancer cell line, OVCAR3-FG. Their antitumor activity was further enhanced in the presence of 5-OP-RU (37). This enhancement highlights the dual activation mechanism for CAR MAIT cells, leveraging both CAR-dependent and MR1-restricted αβTCR-dependent pathways. The study also delved into the performance of mesothelin-CAR MAIT cells within a complex 3D organoid model, incorporating tumor cells, TAMs, and T cells. Remarkably, the mesothelin-CAR MAIT cells maintained their activation and cytotoxicity even in the presence of TAMs, showing their unique ability to counteract M2 macrophages (37). Overall, these findings reinforce the versatility of CAR MAIT cells capable of exerting cytotoxicity through both CAR-dependent and TCR-dependent mechanisms, and their efficacy may be preserved even amidst an immunosuppressive TME (**Figure 2**).

#### 2.2.3 Challenges & solutions

Preclinical *in vivo* investigations concerning CAR MAIT cells remain sparse, many aspects of their potential use in therapy are not fully understood. The detailed safety profiles, the capacity of tumor infiltration, and whether CAR MAIT cells maintain their effector memory subset *in vivo* are key questions that remain unanswered. The *in vivo* persistence of these CAR MAIT cells has yet been extensively studied. There is an imperative need for more researches to elucidate the functionality and safety of CAR MAIT cells. As of now, no clinical trials have been initiated to explore the use of CAR MAIT cells in treatment (**Table 1**).

### 2.3 CAR γδ T cells

#### 2.3.1 Properties and advantages

**Figure 3:**
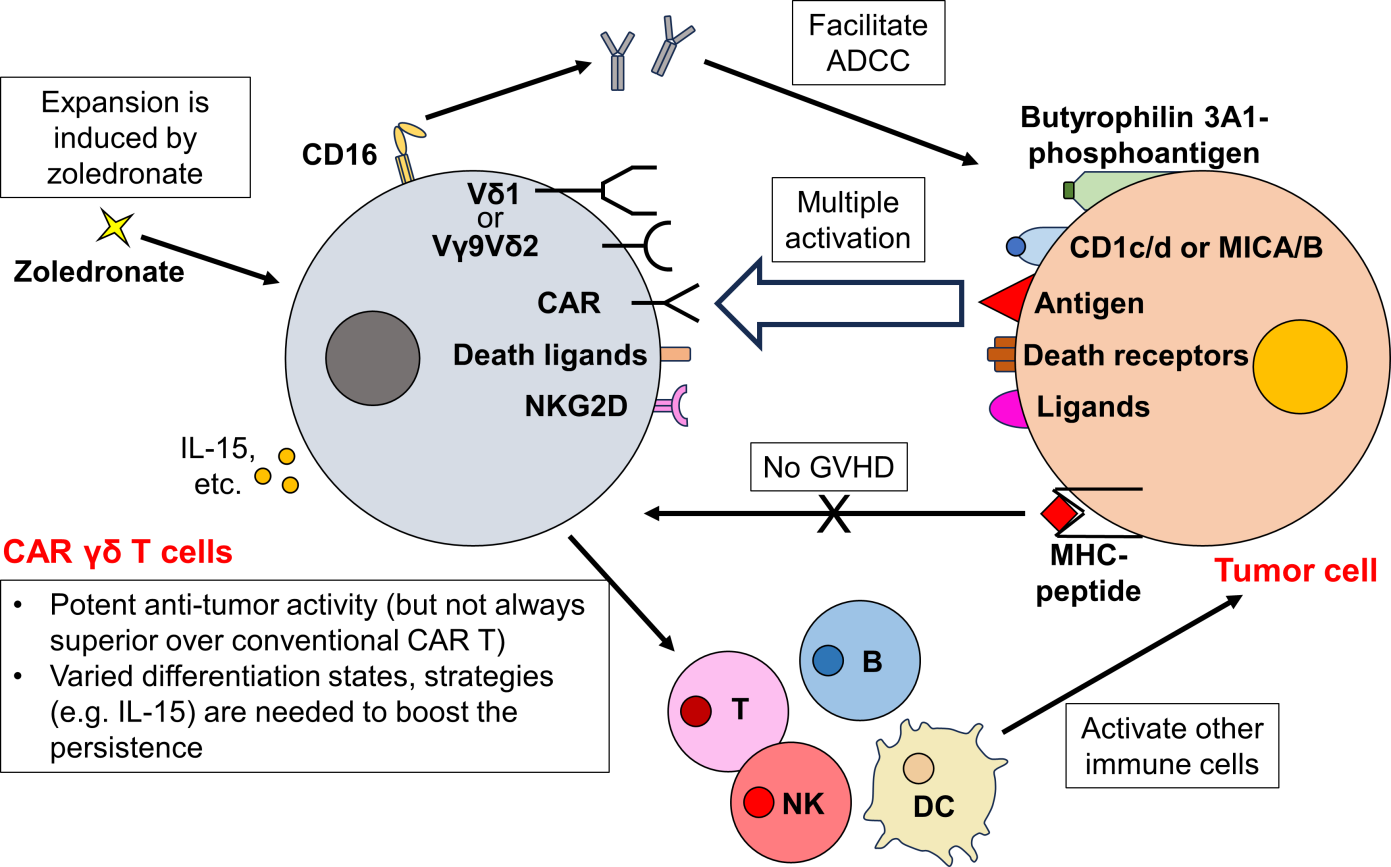
CAR γδ T cells orchestrate a complex immune response and exhibit potent antitumor activity. Besides the traditional CAR-dependent pathway, CAR γδ T cells can target tumor cells through multiple CAR-independent pathways. While Vγ9Vδ2 TCR recognizes phosphoantigens bound by butyrophilin 3A1, Vδ1 TCR targets antigens presented by CD1c/d or MHC class I-like MICA/B. Upon TCR engagement, CAR γδ T cells exert potent cytotoxicity towards tumor cells. Furthermore, CAR γδ T cells also kill tumor cells by expressing death ligands (TRAIL and FasL) and NK receptors (NKG2D). They also upregulate CD16 to eliminate tumor cells via ADCC. As these activation mechanisms are all MHC-independent, the risk of CAR γδ T cells to incur GVHD is low. After activation, CAR γδ T cells further boost the activity of other immune cells, orchestrating a complex antitumor immune response. Although the *ex vivo* and *in vivo* expansion of CAR γδ T cells can be easily achieved through zoledronate administration, the persistence of CAR γδ T cell therapy remains to be determined. Different subsets of CAR γδ T cells reside in different states of differentiation after CAR transduction and expansion, further strategies (e.g. IL-15 administration) are required to ensure a long-term efficacy of CAR γδ T cells.

Gamma delta (γδ) T cells represent a unique subset of T cells characterized by their TCR γ and δ chains. They exhibit both innate and adaptive immune characteristics, including antibody-dependent cellular cytotoxicity (ADCC), direct cytotoxic effects, and antigen presentation (65, 66). Depending on the specific γ and δ chains they express, γδ T cells can be further classified. The predominant γδ T cell subtype in human peripheral blood expresses Vγ9 and Vδ2, hence they are termed Vγ9Vδ2 T cells (67). The Vγ9Vδ2 TCR identifies phosphoantigens, which are small alkyl diphosphates synthesized by exogenous pathogens and diverse tumor cells. These phosphoantigens are presented via butyrophilin 3A1, instead of the conventional MHC (68–70). In contrast, Vδ1 T cells, predominantly resides in tissues, detect antigens presented by CD1c/d or the MHC class I-like molecules, MICA/B (67). Despite their differences in TCR engagement, both Vγ9Vδ2 and Vδ1 T cells exert potent cytotoxic activity against tumor cells (71). Additionally, γδ T cells can target tumor cells by employing mechanisms like engaging death ligands [e.g. TNF-related apoptosis-inducing ligand (TRAIL) and Fas ligand (FasL)] and NK receptors (e.g. NKG2D) (72). Beyond direct cytotoxicity, γδ T cells can upregulate CD16 and engage tumor cells through ADCC (72). Moreover, γδ T cells play an important role in orchestrating the immune response. Activated Vγ9Vδ2 T cells can function as professional APCs, enabling the cross-presentation of antigens to a broad range of immune cells (73–75). Furthermore, they also enhance NK cell-mediated cytotoxicity (76), promote dendritic cell maturation (77, 78), and aid in B cell antibody secretion (79) (**Figure 3**).

Similar to other T cell subtypes discussed, γδ T cells recognize antigens via MHC-independent pathways. Therefore, the primary benefit of deriving CAR cells from γδ T cells is their potential to manifest increased antitumor responses beyond CAR-dependent activity. Additionally, since γδ T cell activation is MHC-independent, these cells are less likely to cause GVHD (80), allowing potential allogeneic application. Furthermore, the tissue-resident nature of Vδ1 T cells might offer enhanced tissue-targeting and tumor infiltration abilities. Notably, increased tumor infiltration by γδ T cells has been correlated with favorable prognostic outcomes (81). Moreover, γδ T cells are advantageous due to their ease of expansion, both *ex vivo* (during the manufacturing process) and *in vivo* following therapeutic administration. This proliferation can be effectively stimulated using amino bisphosphonates such as zoledronate (82–84) (**Figure 3**).

Although different subsets of γδ T cell are enriched in various tissues, PBMCs are still used as the starting materials for CAR γδ T cell manufacturing. Unlike NKT cells and MAIT cells, γδ T cells within PBMCs can be expanded before isolation. For example, CD3^+^Vδ2^+^ T cells consist only about 1.3% of PBMCs. After 10 days of expansion utilizing amino bisphosphonates and various cytokines (i.e. IL-2, IL-7 and/or IL-15), the purity of Vγ9Vδ2 T cells within cultured PBMCs reaches over 90%, making them suitable for CAR Vγ9Vδ2 T cells generation (83, 84). Similarly, although Vδ1 T cells represent only 0.2-1% of PBMCs (85), they can be efficiently expanded using agonistic anti-Vδ1 antibody for CAR cell production (86, 87). Additionally, αβTCR-depletion can be performed to further increase the purity before or after cell expansion for generating CAR cells from bulk γδ T cells [around 3.4% of CD3^+^ cells in PBMCs (88)] or their subsets (88, 89).

#### 2.3.2 Current study

Researchers have developed a variety of CAR products from γδ T cells or their specific subtypes. For instance, Rozenbaum et al. engineered CD19-CAR γδ T cells from PBMCs, which demonstrated potent cytotoxicity against both CD19^+^ and CD19^-^ leukemia cells (88). This capability to target CD19^-^ cells suggests that CAR γδ T cells could offer a novel solution for overcoming antigen-loss relapses. Intriguingly, the cytotoxic activity of these cells was further enhanced by zoledronate. Deniger et al. developed another CD19-CAR γδ T cells demonstrating both CAR-specific and TCR-dependent cytotoxicity, effective against CD19^+^ tumor cells both *in vitro* and *in vivo* (90). Similarly, GD2-CAR γδ T cells, including both bulk population and individual subsets, showed potent cytotoxic effects against GD2^+^ neuroblastoma cell lines (91). Remarkably, upon expansion and activation, these GD2-CAR γδ T cells exhibited properties akin to professional APCs, facilitating CAR-independent tumor cell eradication. Further developments in this field include CD123-CAR Vδ1 T cells against acute myeloid leukemia (AML) (89), carcinoembryonic antigen (CEA)-CAR Vγ9Vδ2 T cells against xenograft mouse model with CEA^+^ tumor (83), B7H3-CAR Vγ9Vδ2 T cells against glioblastoma (84), CD20-CAR Vγ9Vδ2 T cells with conjugated rituximab against B cell lymphoma (92), and HLA-G and PD-L1 multi-specific CAR Vδ2 T cells with the ability to secrete PD-L1/CD3ϵ bispecific T cell engagers (BiTEs, which recruit bystander T cells) against solid tumors (93).

Furthermore, the exploration of CAR γδ T cells in allogeneic setting is increasingly recognized. Makkouk et al. demonstrated the promise of this approach by engineering Glypican-3 (GPC-3)-CAR Vδ1 T cells (87). In a subcutaneous hepatocellular carcinoma xenograft mouse model, these CAR Vδ1 T cells demonstrated enhanced tumor migration and eradication capabilities without eliciting xenograft GVHD. Similarly, Nishimoto et al. observed that allogeneic CD20-CAR Vδ1 T cells displayed enhanced antitumor capabilities without inducing xenograft GVHD (86). Lee et al. advanced this concept by purposely selecting Vγ9Vδ2 T cell donors with high CD16 expression to enhance ADCC (94). The resulting mesothelin-CAR Vγ9Vδ2 T cells exhibited potent antitumor ability in mice intraperitoneal and subcutaneous ovarian cancer models without any signs of GVHD, while all mice treated with conventional CAR T cells died shortly from GVHD. Notably, these CAR Vγ9Vδ2 T cells were also capable of targeting TAMs *in vitro*, suggesting their potential ability of remodeling the TME (94).

Issues like T cell aplasia and fratricide (i.e. CAR T cells attack each other) usually occur in targeting T cell malignancies with conventional CAR T cell therapy. This is because the targeted antigens are regularly expressed on normal T cells and CAR T cells themselves (95). CAR γδ T cells offer an innovative solution to these problems through their MHC-independent cytotoxic mechanisms. Fleischer et al. highlighted this advantage by expressing CD5-targeted non-signaling CARs (NSCARs) in γδ T cells. Hence, the intrinsic cytotoxicity of these cells are redirected towards CD5^+^ T cell acute lymphoblastic leukemia (T-ALL) cell lines (although such antitumor activity is much weaker when compared to CD19-NSCAR γδ T cells against CD19^+^ B-ALL cell lines) (96). Given that CAR NKT cells and CAR MAIT cells also possess inherent MHC-independent antitumor capabilities, exploring their potential could broaden the therapeutic options for these challenging malignancies.

#### 2.3.3 Challenges & solutions

While numerous preclinical studies highlight the potential of CAR γδ T cells in cancer therapy, yet their brief persistence poses a significant challenge. Rozenbaum et al. have shown a rapid decline in CAR γδ T cell numbers, noticeable just three days after injection, in stark contrast to the robust expansion observed with conventional CAR T cells (88). This limited persistence could be attributed to the inherent lack of alloreactivity in CAR γδ T cells. Makkouk et al. further emphasize this difference, revealing that while traditional CAR T cells exhibit substantial *in vivo* expansion, CAR Vδ1 T cells do not (87). Moreover, the persistence and propensity for exhaustion vary across CAR γδ T cell subtypes. Specifically, while Vδ1 T cells predominantly exhibit a naïve phenotype in PBMCs, Vδ2 T cells are mostly characterized as effector memory T cells (91). After CAR transduction and expansion, a significant number of CAR Vδ1 T cells retain their naïve state. This is in contrast to CAR Vδ2 T cells, which tend to differentiate further and display signs of exhaustion (86, 91). It’s well-established that CAR T cells with a more naïve or less differentiated memory phenotype tend to persist longer and manifest an enhanced functional profile (97, 98). Given the observed differences, it’s reasonable to suggest that CAR Vδ1 T cells may offer better *in vivo* longevity compared to CAR Vδ2 T cells. Indeed, this hypothesis is supported by several studies. For instance, Wang et al. reported that the effectiveness of CEA-CAR Vγ9Vδ2 T cells was limited in duration (83). Efforts to enhance the *in vivo* persistence of CAR T cells include the administration of IL-15 and the modulation of intracellular signaling pathways (44). Similar strategies are now being applied to CAR γδ T cells, aiming to extend their persistence (87, 89, 94, 99, 100) (**Figure 3**). For example, Lee et al. engineered IL-15 into their CD16^high^ mesothelin-CAR Vγ9Vδ2 T cells (MCAR15-Vδ2T cells) (94). In mice models of intraperitoneal ovarian cancer, MCAR15-Vδ2T cells exhibited superior tumor control and prolonged persistence in the tumor and organs at day 57 (as compared to normal CAR Vγ9Vδ2 T cells without IL-15). Moreover, all 5/5 mice survived to day 180 with complete remission in MCAR15-Vδ2T cell group, while 3/5 mice died of relapse in normal CAR Vγ9Vδ2 T cell group (94).

Beyond the issue of limited persistence, CAR γδ T cells face several additional challenges that necessitate further investigation. One significant concern is their *in vivo* expansion capacity, which typically does not match that of conventional CAR T cells (87). Consequently, while CAR γδ T cells are theoretically predisposed to migrate to peripheral tissues, questions have been raised about their ability to accumulate in tumors in significant numbers. Moreover, data from *in vitro* transwell migration assays do not demonstrate significant differences in the migratory capacities of CAR Vδ1 T cells, CAR Vδ2 T cells, and conventional CAR T cells (91). This suggests that the superior ability of CAR γδ T cells to penetrate solid tumors requires more rigorous validation. When it comes to cytotoxic performance, CAR γδ T cells do not consistently outperform conventional CAR T cells. In certain instances, their efficacy is even surpassed by conventional CAR T cells (88, 91, 100). Additionally, the intrinsic antitumor responses of γδ T cells vary across different types of tumors, with only moderate activity observed against certain cancers such as ALL and non-Hodgkin lymphoma (NHL) (101, 102). For example, NSCAR γδ T cells showed higher activity against B-ALL cell lines than T-ALL cell lines (96). Thus, CAR γδ T cells might be more effective against specific cancer types, potentially limiting their universal application in cancer therapy (**Table 1**).

#### 2.3.4 Ongoing clinical trials

Several clinical trials are currently underway. In study NCT04735471, the previously mentioned allogeneic CD20-CAR Vδ1 T cells have shown favorable tolerance in lymphoma patients. Of the six participants, four achieved complete remission, with no incidence of GVHD or severe adverse reactions recorded (103). Additionally, there are three ongoing phase I trials: NCT04107142 aims to evaluate the safety and efficacy of haploidentical or allogeneic NKGD2DL-specific CAR γδ T cells in patients with relapsed or refractory solid tumors; NCT02656147 assesses allogeneic CD19-CAR γδ T cells in patients diagnosed with high-risk or r/r B cell malignancies; and NCT04702841 examines CD7-CAR γδ T cells for patients with relapsed or refractory CD7^+^ T cell malignancies (**Table 2**). As of now, results from these trials remain unpublished.

### 2.4 CAR CIK cells

#### 2.4.1 Properties and advantages

Cytokine-induced killer (CIK) cells represent a heterogeneous group of T-NK killer lymphocytes. They are derived *ex vivo* from PBMCs, primarily from the CD3^+^CD56^-^CD8^+^ T cell subsets, after extensive culture in the presence of anti-CD3 antibodies, IFN-γ, and IL-2 (104). After 2-3 weeks of cultivation, the predominant phenotype among the expanded cells is CD3^+^CD56^+^, with a smaller fraction of CD3^+^CD56^-^ cells (104). Intriguingly, these cells exhibit markers of NK cells (e.g. activating receptor NKG2D) to a variable extent, while also retaining hallmark T cell markers (105). This combination of markers empowers CIK cells with TCR-mediated cytotoxicity and MHC-independent NK cell-like functions (106). CIK cells demonstrate potent antitumor efficacy against various tumor cells. Activated CIK cells also upregulate the expression of FasL and perforin, partially mediated by NKG2D, facilitating tumor elimination (107). Additionally, CIK cells release a variety of pro-inflammatory cytokines, enhancing systemic immune responses against tumors (104).

As the predominant cytotoxic effector phenotype within CIK cells is CD3^+^CD56^+^, some studies suggest these cells could be categorized as NKT cells. However, it’s essential to clarify that these are not the previously mentioned iNKT cells, as CIK cells do not depend on CD1d for their activation (108, 109). Despite this distinction, CIK cells’ MHC-independent cytotoxic activity aligns them with other T cell subtypes discussed earlier. Consequently, CAR CIK cells are believed to offer similar benefits, including robust endogenous antitumor activity, enhanced safety profiles, and reduced risk of GVHD (110, 111). Furthermore, the nature of CIK cells ensures an ample source for CAR transduction. In addition, CIK cells have shown an ability to navigate to tumor sites following infusion, although a detailed evaluation of their tumor infiltration effectiveness remains to be conducted (110, 112).

#### 2.4.2 Current study

To date, numerous CAR CIK cell therapies utilizing first-, second-, or third-generation CAR constructs have been developed for various hematologic malignancies and solid tumors (113–118). A quintessential example is the human epidermal growth factor receptor 2 (HER2)-CAR CIK cells, created by Merker et al. These cells have demonstrated potent antitumor activity against rhabdomyosarcoma xenograft models without severe adverse effects (119). Yet, it’s worth noting that despite suggestions that HER2-CAR CIK cells have better tissue migration and persistence than wild-type CIK cells, histological evaluations using CD3 staining showed these cells to be scarce in most tissues. Other CAR CIK cells have demonstrated similarly potent cytotoxic abilities as well. For instance, 5T4-CAR CIK cells have been effective in eliminating nasopharyngeal carcinoma stem cell-like cells via both CAR-dependent and NKG2D-mediated CAR-independent mechanisms (120). Additionally, CD123-CAR CIK cells also exhibit inherent cytotoxic effects on CD123^-^ cells, complementing their CD123-targeted capabilities. Importantly, CD123-CAR CIK cells do not significantly increase their immunostimulatory cytokine release upon CARactivation compared to regular CIK cell activation. This suggests that CAR CIK cells might not trigger severe adverse effects (117). Research has also explored targeting both the leukemic cell marker CD33 and the mesenchymal stromal cell marker CD146 using CAR CIK cells (121). Delving deeper, this study has shown that mesenchymal stromal cells can attenuate the long-term activity of single-targeted CAR CIK cells. This reveals that CAR CIK cells, like CAR T cells, are vulnerable to the immunosuppressive TME.

#### 2.4.3 Challenges & solutions

The therapeutic efficacy of CIK cell therapy is considered somewhat limited, leading to suggestions that a large number of CIK cells is required for optimal tumor elimination (122, 123). Additionally, the *in vivo* antitumor activity of CAR CIK cells is reported to be less than that of CAR T cells (124) [Despite this, one study also proposed that CAR CIK cells and conventional CAR T cells have comparable *in vitro* cytotoxicity (125)]. This reduced efficacy of CAR CIK cells is partially due to their diminished persistence. It has been reported that CSPG4-CAR CIK cells can only control tumor (i.e. soft tissue sarcoma) growth for 2 weeks (126), indicating a significantly low persistence of CAR CIK cells. Generally, CIK cells are characterized as terminally differentiated effector memory T cells (TEMRA). These cells have limited proliferative ability and are prone to apoptosis (127). Such profile is anticipated, given that CIK cells emerge from extensive *ex vivo* expansion. Notably, IL-15 is known to promote T cell activation and proliferation (128, 129). Thus, the inclusion of IL-15 during the *ex vivo* cultivation process can markedly enhance the cytotoxic potential of CAR CIK cells (115). Interestingly, CAR CIK-like cells can be induced directly, bypassing the need of extensive *ex vivo* expansion and differentiation. This helps prolong the persistence of CAR cells. Hombach et al. integrated IL-12 into the exodomain of CAR (130). Upon activation, this IL-12-CAR then reprograms CD8^+^ T cells into heterogenous human leukocyte antigen E (HLA-E)-restricted NK-like cells. A major subset of these cells, characterized by the CD8^+^CD56^+^CD62L^high^ expression, closely resembles CIK cells. *In vitro* test of these CEA-targeted IL-12-CAR T cells exert both antigen-dependent and -independent cytotoxicity against tumor cells. Importantly, such cytotoxicity persists upon repeated antigen stimulation (for 7 days). In contrast, the activity of conventional CAR T cells declines under the same condition. As a result, CEA-targeted IL-12-CAR T cells show superior antitumor activity (than conventional CAR T cells) in mice subcutaneous tumor models (130).

The proper design of CAR constructs plays a crucial role in ensuring the sustained efficacy of CAR CIK cells, similar to what is required for CAR T cells. Hombach et al. conducted a comparative analysis on the activity of CAR CIK cell using three generations of CAR constructs: the first-generation CD3ζ-CAR, the second-generation CD28-CD3ζ-CAR, and the third-generation CD28-CD3ζ-OX40 CAR (131). Surprisingly, the addition of costimulatory domains, such as CD28 alone or in combination with OX40, enhanced the initial activation and short-term activity of CAR CIK cells (122, 131). However, the synergistic costimulation through both CD28 and OX40 led to accelerated maturation of these terminal CAR CIK cells. This acceleration promoted activation-induced cell death (AICD). As a result, this further maturation correspondingly attenuated the NKG2D-mediated MHC-independent cytotoxic activity of the CAR CIK cells. In contrast, CD28-mediated costimulation alone did not push the cells to mature more than those with the CD3ζ-CAR (131). Consequently, the long-term antitumor activity of CD28-CD3ζ-OX40 CAR CIK cells was significantly lower than that of the CD28-CD3ζ-CAR (131). Nonetheless, another study reported that the third-generation CD28-4-1BB-CD3ζ-CAR CIK cells showed superior long-term antitumor efficacy compared to both the first-generation CD3ζ-CAR and the second-generation CD28-CD3ζ-CAR (124). This underscores the significance of costimulatory domain optimization in engineering CAR CIK cells.

One critical aspect of CAR CIK cells is their potential toxicities. Although most CIK cells exhibit the CD3^+^CD56^+^ phenotype, the CD3^+^CD56^−^ subset of CIK cells also plays a significant role in cytotoxicity against tumor cells (132). Following CAR integration, it’s noteworthy that the majority of CAR CIK cells retain their TCRα/β signatures (133). Despite this, almost all preclinical studies report no or low adverse effects from CAR CIK cells. Another aspect requiring further investigation is the tumor infiltration ability of CAR CIK cells. As previously elucidated, the presence of CAR CIK cells in various tissues is limited (119). To improve the migration of CD33-CAR CIK cells to the bone marrow, Biondi et al. overexpressed CXCR4 in these CAR CIK cells. As a result, these CXCR4-overexpressing CAR CIK cells demonstrated not only an enhanced affinity for the bone marrow environment but also increased antileukemic activity (134) (**Table 1**).

#### 2.4.4 Ongoing clinical trials

NCT03389035 is a phase I/II clinical trial evaluating the safety and efficacy of allogeneic CD19-CAR CIK cells. These cells are engineered using the non-viral vector, the Sleeping Beauty transposon. Within the cohort of 27 patients with r/r ALL post-HSCT (including a group of 6 patients undergoing compassionate-use treatment), 18 patients achieved a complete response. The overall survival rate was 71.4% over a median follow-up of 2.8 years. No GVHD were reported. However, 11 patients experienced CRS or ICANS (135). Concurrently, another clinical trial, NCT05869279, is exploring the use of haploidentical CD19-CAR CIK cells against B cell NHL or CLL, though its outcomes have not been reported (**Table 2**).

## 3 CAR cells generated from innate immune cells

In addition to T cells, innate immune cells offer a promising avenue for CAR product. Owing to their capacity to recognize and eliminate targets in an MHC-independent manner, CAR cells derived from innate cells may preserve this characteristic. Consequently, these CAR cells might demonstrate enhanced antitumor activity, not only from their engineered CAR-induced cytotoxicity but also from their inherent functions, akin to the various T cell subtypes discussed earlier. Currently, CAR NK cells, CAR macrophages and CAR neutrophils are garnering significant interest.

### 3.1 CAR NK cells

#### 3.1.1 Properties and advantages

CAR NK cells are probably the second most recognized CAR-associated cell products. As components of the innate immune system, NK cells depend on a delicate balance of activating and inhibitory signals to target tumor cells. This is achieved through Perforin/Granzyme B pathways or apoptosis mechanisms (136). Numerous reviews have already delved into the research and clinical trials of CAR NK cells (136–139). As this review predominantly focuses on novel CAR-related treatments, only a brief overview of CAR NK cells is provided here.

CAR NK cells possess the innate ability to retain their endogenous activation receptors (137). This attribute makes them a powerful tool to reduce relapse due to loss of antigen. This feature also ensures CAR NK cell activity, even upon CAR downregulation. Furthermore, NK cells do not secrete key cytokines, such as IL-1 and IL-6, which are known to trigger CRS (137). Clinical trials have confirmed the safety of CAR NK cells, reporting minimal to no side effects. These trials also demonstrate their effectiveness against both solid and hematological malignancies (140–142). Another advantage of NK cell-based CAR therapies is their minimal alloreactivity (143), facilitating the development of allogenic CAR NK cell products. Sources of these non-autologous CAR NK cells include NK cell lines (141, 144), cord blood sources (142, 145), allogeneic NK cells (140), and allogeneic iPSC-derived NK cells (146).

#### 3.1.2 Current study

Currently, the research focus of CAR NK cells is to explore strategies that can further enhance their antitumor activity. One approach to enhance the ability of CAR NK cells to infiltrate tumors is through the overexpression of various chemokines. For instance, by overexpressing CXCR1, NKG2D-CAR NK cells have been shown to migrate and infiltrate solid tumors in mouse models of ovarian cancer (147). In order to develop optimal and specific CAR constructs for NK cells, Li et al. conducted a screening of various CAR constructs that contained different signaling domains. They found that a CAR construct with the transmembrane domain of NKG2D, the 2B4 co-stimulatory domain, and the CD3ζ signaling domain was able to induce strong antigen-specific NK cell activity (148). To further enhance the antitumor activity, the researchers deleted cytokine-inducible Src homology 2-containing protein (CIS), the key negative regulator of IL-15 signaling, in CAR NK cells (149). As a result, the activity of IL-15-secreting CAR NK cells was enhanced through the Akt/mTORC1 and c-MYC pathways, which promote aerobic glycolysis.

#### 3.1.3 Challenges & solutions

The *in vivo* persistence of CAR NK cells is a significant challenge for their broader clinical application. Notably, limited expansion of CAR NK cells has been observed in patients demonstrating suboptimal therapeutic outcomes (142). Addressing this issue may involve strategies used to enhance CAR T cells. For instance, integrating IL-15 into CAR T cell therapies has significantly improved persistence and proliferation in various murine studies (150, 151). This approach has shown clinical benefits in a patient with B-ALL after failures with conventional and CAR T-cell treatments (152). Moreover, the costimulatory signals MyD88/CD40 have been found to boost CAR T cell proliferation and expansion (153, 154). Inspired by these findings, efforts to prolong CAR NK cell persistence have employed IL-15 signaling or MyD88/CD40 pathways (149, 155). Genetic engineering of CAR NK cells offers another strategy to enhance their therapeutic effectiveness (156) (**Table 1**).

#### 3.1.4 Ongoing/completed clinical trials

Currently, there are around 70 ongoing or completed clinical trials evaluating the effectiveness of CAR NK cells against tumors. A list of CAR NK cell-related clinical trials can be found in a recently published review (157). NCT02944162 is one of the earliest first-in-human phase I clinical trials of CAR NK cells, which assessed the safety of CD33-CAR NK cells in patients with r/r AML. Although the infusion of CD33-CAR NK cells at a dose of up to 5×10^9^ cells per patient was deemed safe, all three enrolled patients either showed no response or relapsed at 15 months/5 years post-treatment (141). While this result are not satisfactory, other studies may yield better outcomes (although their follow-up period may not be long enough). For instance, in the phase I trial (NCT04623944), allogeneic NKG2D ligand-directed CAR NK cells exhibited a complete response rate of 67% in patients with r/r AML (3/6 achieved a complete response with hematologic recovery, and 1/6 had a complete response with incomplete hematologic recovery) (158). Similarly, in another trial (NCT05020678), 8 out of 14 patients with r/r NHL achieved a complete response after receiving allogeneic CD19-CAR NK cells (although 3 patients with indolent lymphoma experienced relapse after more than 6 months) (159). However, almost all 5 patients with B cell-driven leukemia (ALL and CLL) enrolled in the same trial did not show any response, except for one with CLL achieved stable disease. Nevertheless, no dose limiting toxicities, neurotoxicity, GVHD or long-lasting cytokine release syndrome (beyond 24 hours) were reported in all patients, further indicating the safety of CAR NK cells. More recent trials are assessing the efficacy of CAR NK cells with different modifications. For example, the phase I/IIa study NCT05410717 is evaluating the safety and efficacy of Claudin6, Glypican-3 (GPC3), mesothelin, or AXL-directed CAR NK cells in patients with Claudin6, GPC3, mesothelin, or AXL-positive advanced solid tumors. These CAR NK cells can secrete IL-7/CCL19 and/or scFvs against PD1/CTLA4/Lag3 to enhance their activity and prevent immunosuppression. In NCT05703854, CD70-CAR NK cells engineered with IL-15 are being assessed in patients with advanced renal cell carcinoma, mesothelioma and osteosarcoma as well. It is expected that these modified CAR NK cells will exhibit improved efficacy.

### 3.2 CAR macrophages

#### 3.2.1 Properties and advantages

**Figure 4:**
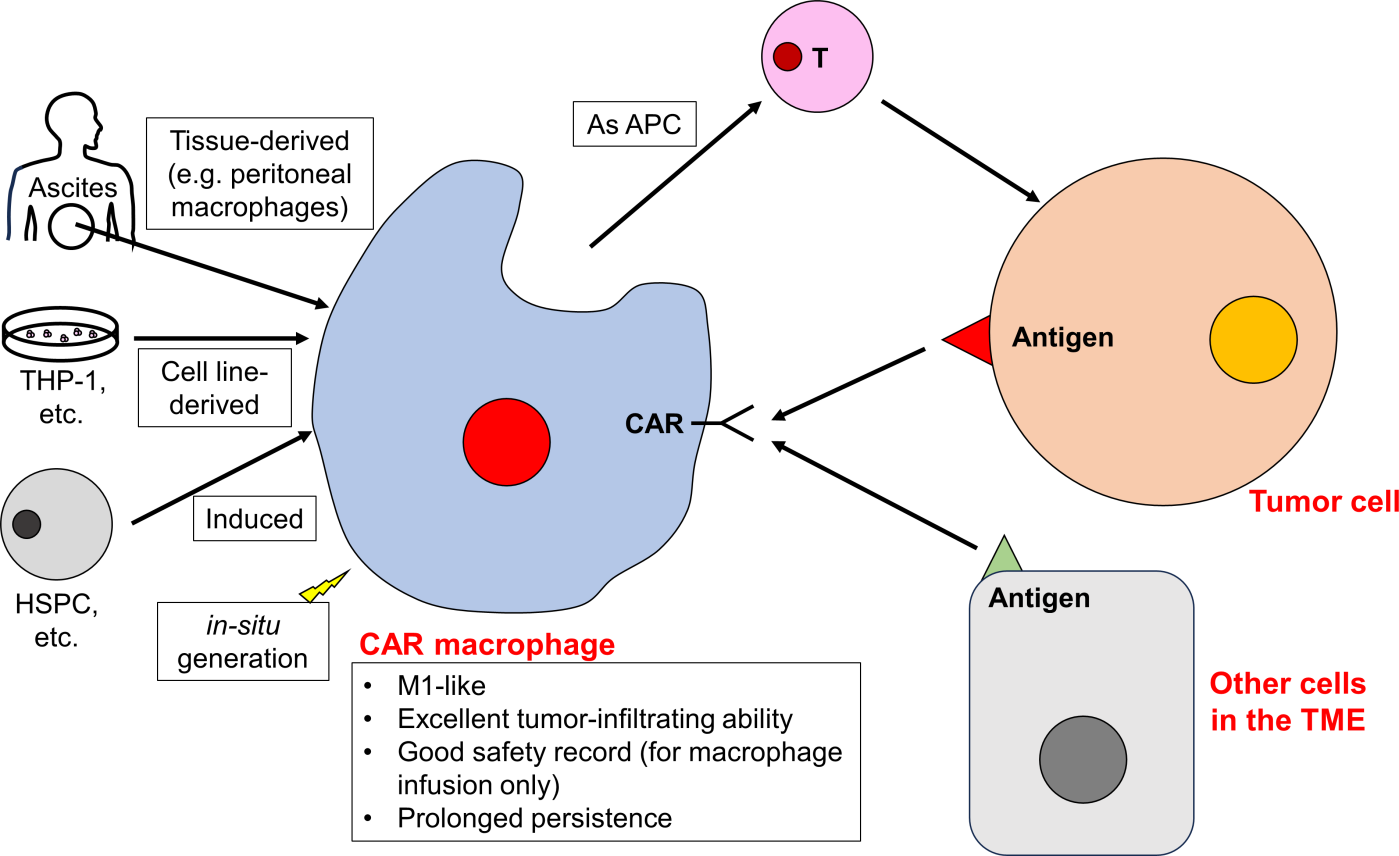
CAR macrophages play a pivotal role in reshaping the TME. CAR macrophages can be developed from tissue-resident macrophages, cell lines, differentiated progenitor cells, or generated *in situ*. CAR macrophages remain to be M1-like and exhibit excellent tumor-infiltrating ability, and they are able to target tumor cells and reshape the TME (including targeting other cells in the TME and facilitating the function of other immune cells).

As the central regulator of innate immunity, macrophages are highly plastic innate immune cells with a broad spectrum of effector functions. These include direct phagocytosis, antigen presentation, and modulation of the TME (160). Introducing CAR constructs into macrophages aims to direct their phagocytic activity specifically towards cancer cells. TAMs comprise a substantial number of tumor-infiltrating immune cells (161, 162), and this innate trait makes CAR macrophages promising candidates for penetrating solid tumors. Historically, the use of macrophages has been considered safe (163). This suggests that CAR macrophages are likely to be well-tolerated, with a low risk of adverse effects (**Figure 4**).

Macrophages can be classified into two main polarization states: the classically activated, proinflammatory M1 and the alternatively activated, anti-inflammatory M2 (164). Within tumors, TAMs predominantly resemble the M2 phenotype (164). These TAMs contribute to an immunosuppressive TME by recruiting regulatory T cells (Tregs) and myeloid-derived suppressor cells (MDSCs), as well as suppressing cytotoxic effector cells (165). Given the significant role of macrophages in shaping immune microenvironments, there are efforts to introduce genetically engineered CAR macrophages, especially M1-like macrophages, into tumors.

While many CAR cells are derived from PBMCs due to their abundance and expandability, macrophages in PBMCs are relatively rare. This rarity makes direct developing CAR macrophages from PBMCs challenging. As a result, researchers have turned to alternative sources for macrophages, including tissue-derived macrophages, established macrophage cell lines, precursor or stem cell-derived macrophages, and the innovative concept of *in situ* CAR macrophage generation. For instance, ascites, which is rich in peritoneal macrophages, has emerged as a potential source (166). In murine tumor models, the mouse macrophage cell line, RAW 264.7, has been used to create CAR macrophages (167–169). In human settings, the THP-1 cell line, a macrophage/monocyte derivative, has been utilized (170, 171). Macrophages can also be induced from hematopoietic stem and progenitor cells (172–176), human pluripotent stem cells (177), and iPSCs (175, 178–180). In addition, there is interest in the *in-situ* generation of CAR macrophages using nanoparticles as well (181–184). These nanoparticles can induce CAR expression, direct phagocytic activity towards tumor cells, and prompt a shift from M2-like TAMs to M1-like macrophages. This shift creates a tumor suppressive immune microenvironment (**Figure 4**). With *in situ* CAR macrophages production, there are no concerns about GVHD. Additionally, this method allows CAR macrophages to cross the blood-brain barrier and further enhance tumor infiltration. For example, Gao et al. locoregionally edited intratumoral macrophages (of brainstem gliomas) into M1-like HER2-specific CAR macrophages (182).

#### 3.2.2 Current study

Due to their unique advantages in tumor infiltration, several CAR macrophages have been designed specifically to target solid tumors. These engineered macrophages exhibit dual capabilities of phagocytosis and immunomodulation. Notably, HER2-specific CAR macrophages targeting various tumor models have attracted significant attention (166, 170, 172, 173, 182). Dong et al. developed HER2-CAR macrophages for gastric cancers, utilizing human peritoneal macrophages (166). Upon activation, these CAR macrophages shift towards an M1-like phenotype with enhanced antigen-specific phagocytosis and antigen presentation to promote T cell proliferation. In multiple gastric cancer models, these CAR macrophages inhibited tumor growth without significant toxicity. They also exhibit synergistic effects with first-line chemotherapy (166). Klichinsky et al. engineered another HER2-CAR macrophage with an M1-like phenotype. It was derived from the THP-1 lineage or differentiated monocytes. This design aimed to amplify the endogenous immune response and increase the specificity of phagocytic activity (170). Characterized by prolonged persistence, these CAR macrophages show minimal toxicity, including rare occurrence of on-target, off-tumor toxicity (170). The M1 polarization of these HER2-CAR macrophages can be pre-conditioned *in vitro* using LPS and IFN-γ prior to therapeutic infusion, thereby further enhancing their antitumor activity (173). Remarkably, HER2-CAR macrophages can prevent antigen-negative relapses, suggesting their potential long-term activity (172). In another study, Chen et al. demonstrated that HER2-CAR macrophages promote the proliferation and activation of CD8^+^ cytotoxic T lymphocytes. They also remodel the overall macrophages within tumors (including the CAR macrophages themselves and TAMs) into an M1-like phenotype (171). It is proposed that the interaction between CAR macrophages and other immune cells in the TME may contribute to maintaining the M1-like phenotype of CAR macrophages (171) (**Figure 4**).

In addition to HER2-CAR macrophages, researchers have developed a variety of other CAR macrophages. Examples include: CD133-CAR macrophages against glioblastoma (184), CD19-CAR macrophages against leukemia cell lines (178), mesothelin-CAR macrophages against ovarian or pancreatic cancer cell lines (178), GD2-CAR macrophages against neuroblastoma (177), GPC3-CAR macrophages against hepatocellular carcinoma (181), anaplastic lymphoma kinase (ALK)-CAR macrophages against neuroblastoma (183), and CEA-CAR macrophages against CEA^+^ tumor cells (CEA is a cell adhesion protein upregulated in various solid tumors) (174).

Beyond macrophages engineered to directly target tumor cells, there is a growing interest in designing CAR macrophages to modulate the TME. For instance, Zhang et al. introduced the HER2-specific CAR-147 macrophage (167). Although the cell targets HER2, the HER2 antibody is linked to CD147, a membrane protein that promotes the expression of Matrix metalloproteinases (MMPs). While these CAR-147 macrophages did not show direct cytotoxicity against tumor cells *in vitro*, they demonstrated significant antitumor efficacy *in vivo*. Their effectiveness is not from direct cellular cytotoxicity but by degrading the tumor extracellular matrix via MMPs. This degradation enhanced T cell infiltration. A notable finding was the reduction of cytokines such as IFNγ, TNFα, and IL-6 in blood following CAR-147 macrophage therapy (167). Another similar approach focused on vascular endothelial growth factor receptor-2 (VEGFR2). VEGFR2 is essential for angiogenesis, and is highly expressed in the vascular endothelial cells of the TME (185). Upon activation, VEGFR2-CAR macrophages adopt an M1-like phenotype and demonstrate potent antitumor activities. Notably, their mechanism does not rely on the direct phagocytosis of tumor cells. Instead, they create an antitumor immune microenvironment to control tumor growth (169). Similarly, CCR7-CAR macrophages target lipid droplet-rich CCR7^+^ immunosuppressive cells within the tumor, with the potential to remodel the immunosuppressive TME (168) (**Figure 4**).

The innate ability of CAR macrophages to boost the function of endogenous T cells suggests they could enhance CAR T cell activity. When co-cultured *in vitro*, CAR macrophages and CAR T cells exhibited remarkable synergy in cytotoxic activity against tumor cells (186). While CAR macrophages support the function of CAR T cells by upregulating costimulatory ligands (CD86 and CD80), CAR T cells also secrete proinflammatory cytokines that drive CAR macrophages towards an M1 polarization. These M1-polarized CAR macrophages further increase the expression of CD86 and CD80, creating a powerful and self-reinforcing loop of CAR cell activation (186). Exploring this synergistic dynamic *in vivo* represents a promising area of research.

#### 3.2.3 Challenges & solutions

A major challenge in using CAR macrophages is the scarcity of macrophages available for CAR macrophages production. Adding to this challenge is the nature of macrophages themselves: they typically exhibit limited proliferation both *in vitro* and *in vivo*, though this observation has been debated (187). These limitations make it difficult to massively produce CAR macrophages, and they may require multiple administrations to maintain therapeutic levels within patients. As a solution, researchers have explored various macrophage sources, as previously mentioned. Yet, concerns regarding the potential tumorigenicity of products derived from progenitor cells, particularly those from iPSCs, pose significant barriers to their clinical utilization (188, 189). It is crucial to rigorously evaluate the safety of CAR macrophages derived from these cells. Furthermore, studies examining the post-infusion *in vivo* phenotype of CAR macrophages are essential, with a particular emphasis on their persistence. Such research could offer key insights into the necessary dosages for CAR macrophage treatments, and also assess the possibility of maintaining prolonged antitumor effects with minimal therapeutic levels.

In addition to persistence, the ability of CAR macrophages to maintain their antitumor phenotype within the TME requires carefully study. While many studies have shown the M1-like polarization of CAR macrophages, these investigations were either conducted *in vitro* or over short period. Given that the TME contains numerous factors that induce TAMs towards an M2-like phenotype (164), it is crucial to verify if CAR macrophages can resist such modulation. This resistance is also essential for the sustained activity of CAR macrophages. Using an adenoviral vector, Ad5f35, Klichinsky et al. successful engineered CAR macrophages that exhibited a persistent M1-like phenotype even when exposed to M2-promoting factors (170). Moreover, Wang et al. identified ACOD1 as a key regulator of the pro-inflammatory M1 state in macrophages using CRISPR screening (180). ACOD1 knockout CAR macrophages derived from iPSCs exhibited enhanced and sustained M1 polarization. These cells had elevated ROS production, phagocytosis and cytotoxicity. In addition, proper CAR construct design might also help CAR macrophages resist M2 polarization. The “second-generation” CAR macrophages, with tandem domains of CD3ζ-TIR-CAR, maintained in M1-polarization due to the TIR domain in a nuclear factor kappa B (NF-κB)-dependent manner (179). However, this *in vitro* experiment lasted only 7 days. Therefore, comprehensive long-term *in vivo* evaluations are still needed.

The introduction of genes into macrophages emerges as another major challenge in CAR macrophage production. Macrophages naturally resist viral transfection, making effective genetic manipulation difficult (170). The aforementioned Ad5f35 is a replication-deficient adenoviral vector designed specifically for hematopoietic cells (190). In addition, Gao et al. generated a chimeric lentiviral vector (HIV-1-Vpx) by packing the HIV-2 accessory protein Vpx into the virus (191). Vpx promotes the degradation of SAMHD1, a myeloid-specific restriction factor that inhibits the virion cycle in macrophages. As a result, HIV-1-Vpx carrying the CAR construct can efficiently infect macrophages. Besides using specific vectors, Dong et al. enhanced the efficacy of lentiviral transduction by pre-treating macrophages with Vitamin D3 and NATE™ (166).

Optimal CAR construct design is crucial. Morrissey et al. select three distinct intracellular domains from phagocytic receptors for their CAR constructs. These included the common γ subunit of Fc receptors (FcRγ, termed CAR-FcRγ; FcR triggers the engulfment of antibody-bound particles), the intracellular domain of Megf10 (designated CAR-Megf10; murine phagocytic receptors for apoptotic cell recognition), and the CD19 cytoplasmic domain (named CAR-PI3K; for the recruitment of p85 subunit of PI3K) (192). While CAR-FcRγ and CAR-Megf1 exhibited robust trogocytosis, they were not effective in whole-cell phagocytosis. In contrast, the CAR-tandem construct, consisting of FcRγ and the CD19 cytoplasmic domain, demonstrated a superior ability for whole cell digestion. Further findings from their research indicated that the inclusion of TCR CD3ζ chain into the CAR construct and CD47 blockade enhanced the phagocytosis of CAR macrophages (192). Using the same CAR constructs, another study showed that these constructs provided CAR macrophages with varied cytotoxic and phagocytic capabilities (186). Additionally, the previously introduced second-generation iPSC-derived M1-polarized CAR macrophages contain tandem domains of CD3ζ-TIR-CAR (179). Upon CAR activation, the TIR domain (via the TLR4 signaling pathway) and the CD3ζ domain (to a lesser extent) both contribute to the production of pro-inflammatory cytokines. They induce M1-polarization and trigger CAR-dependent phagocytosis (179). Lei et al. further observed that these CD3ζ-TIR-CAR macrophages secrete TNF to induce tumor cell apoptosis, and these apoptotic cells are then cleared by CAR macrophages through efferocytosis. (It should be noted that Lei et al. refer to the CAR macrophages with CD3ζ-CAR as the first-generation CAR macrophages, as CD3ζ-CAR is originally designed for CAR T cells. CD3ζ-TIR-CAR is specifically designed for macrophages and is thus called the second-generation CAR. However, as previously mentioned, there are already numerous CAR constructs with different intracellular domains developed for macrophages. By this criterion, these CAR macrophages should also be classified as the second-generation CAR.) Beyond linking to intracellular domains of phagocytic receptors, CARs can incorporate other signaling domains. These include domains for T cell activation or immune microenvironment remodeling, like the CAR-147. There is still no consensus on the most suitable CAR macrophage constructs yet, and the design of CAR macrophage constructs needs optimization based on the purpose of the treatment (**Table 1**).

#### 3.2.4 Ongoing clinical trials

At present, only one clinical trial involving CAR macrophages has been conducted, using the HER2-CAR macrophages developed by Klichinsky et al. (170). This phase I, first-in-human study (NCT04660929) enrolled 7 patients with advanced HER2-overexpressing solid tumors, all of whom had failed prior treatment. The CAR macrophages were generated by differentiating monocytes from mobilized apheresis products. Remarkably, the CAR macrophages were well-tolerated by all participants, with no reports of severe organ damage or on-target, off-tumor toxicities. The study also noted increased tumor infiltration and activation of the TME. This activation was accompanied by improved T cell functions, including enhanced infiltration, proliferation, and activation. However, at 8 weeks, none of the 4 evaluated patients achieved remission: 3 patients had stable disease, and one patient experienced disease progression (193) (**Table 2**).

### 3.3 CAR neutrophils

#### 3.3.1 Properties and advantages

Neutrophils, constituting over 50% of human circulating leukocytes, are abundantly accumulated in various cancers (194). While they can facilitate tumor growth, by fostering angiogenesis and an immunosuppressive TME, they also display antitumor effects due to their high plasticity. Their antitumor activity includes direct cytotoxicity, activation of T cells and trogoptosis (neutrophil-mediated ADCC) (194). Therefore, harnessing the antitumor capabilities of neutrophils through CAR technology offers immense therapeutic potential. As neutrophils and macrophages are both key to innate immunity, the CAR cells derived from them have similar advantages and limitations. These include pronounced tumor infiltration, enhanced antitumor activities based on their intrinsic function, the ability of immunomodulation, a promising safety profile, and the potential for off-the-shelf applications.

#### 3.3.2 Current study

Chang et al. developed chlorotoxin (CLTX)-directed CAR neutrophils for glioblastoma, derived from human pluripotent stem cells (195). These CAR neutrophils demonstrate remarkable mobility and selectively eliminate antigen-bearing tumor cells. They use a multifaceted approach for this: phagocytosis, reactive oxygen species (ROS) generation, and neutrophil extracellular trap formation (a process wherein neutrophils deploy their DNA to capture and eliminate targets). Like macrophages, neutrophils also exhibit antitumor N1 and pro-tumor N2 phenotypes within the hypoxic TME (196). While normal neutrophils shift to N2 phenotype under hypoxia *in vitro*, CLTX-CAR neutrophils remain N1-like under the same conditions (195). This suggests that CLTX-CAR neutrophils might maintain an antitumor N1-like phenotype in the hypoxic TME *in vivo* as well. Moreover, CLTX-CAR neutrophils can carry hypoxia-activated pro-drug to achieve precise drug delivery into glioblastoma (197). Using a similar approach, the same team developed prostate-specific membrane antigen (PSMA)-CAR neutrophil against prostate cancers as well (but have been only tested *in vitro*) (198).

#### 3.3.3 Challenges & solutions

A significant limitation of CAR neutrophils is their brief lifespan, typically just a few days (194). This short existence prevents the direct generation of CAR neutrophils from mature neutrophil. Instead, CAR neutrophils can only be developed from CAR-transduced human pluripotent stem cells or iPSCs. This raises safety concerns about these cells. However, generating neutrophils from CAR-transduced iPSCs bypasses the challenges of editing primary neutrophils’ genomes. Furthermore, the short lifespan of neutrophils also brings up questions about the duration and persistence of their therapeutic effects *in vivo*. This might necessitate repeated infusions for sustained treatment effectiveness. In addition, the ability of CAR neutrophils to persistently maintain the N1 phenotype within the TME needs further study (**Table 1**).

## 4 CAR products generated from alternative sources

Beyond direct derivation from immune cells, there is an innovative approach wherein hematopoietic stem and progenitor cells (HSPCs) are engineered with CAR constructs prior to transplantation. These CAR HSPCs then act as progenitors for a variety of CAR immune cells. Additionally, exosomes secreted by CAR T cells have shown potent cytotoxicity against tumor cells, extending CAR-based therapy beyond cells.

### 4.1 CAR HSPCs

#### 4.1.1 Properties and advantages

HSPCs can be isolated from bone marrow, peripheral blood post G-CSF mobilization, and umbilical cord blood (199). After HSCT, the transplanted HSPCs generate neutrophils within a month. NK cells appear between 1-3 months, T cells emerge after 100 days, and B cells develop within 1-2 years (200). Given their pluripotent nature, HSPCs, as immature multipotent stem cells for all hematopoietic cell lineages, can theoretically give rise to nearly all types of CAR immune cells after transplantation. A key advantage of CAR HSPCs is their potential for sustained antitumor effects, owed to the continuous generation of diverse CAR-immune effectors. Moreover, since HSCT is a cornerstone treatment for many hematological malignancies, processing of HSPCs (CD34^+^ cells) and incorporating CAR HSPCs into the HSCT framework is clinically feasible.

#### 4.1.2 Current study

CD4-CAR HSPCs have been developed for human immunodeficiency virus (HIV) management in both humanized murine and macaque models (201, 202). After transplantation, these CAR HSPCs differentiate into multiple hematopoietic lineages. These cells proliferate across various tissues and maintain their presence for nearly two years. Turning to cancer treatment, De Oliveira et al. demonstrated that CD19-CAR engineered HSPCs can differentiate into myeloid or NK cells *in vitro* (203). When tested *in vivo*, these CAR HSPCs behave like normal HSPCs. Remarkably, they can differentiate into multiple hematopoietic lineages and suppress tumor growth, even at 32 weeks after transplantation. The researchers further introduced a suicide gene, the herpes simplex virus thymidine kinase HSVsr39TK, into these CAR HSPCs (204). This suicide gene does not affect the engraftment, differentiation and antitumor activity of CAR HSPCs, and the differentiated CAR cells in mouse tissues can be eliminated after ganciclovir administration. However, not all gene-modified cells, especially those within bone marrows, can be fully removed. This is probably due to the protective effects of bone marrow. In addition to HSVsr39TK, the authors co-delivered truncated epidermal growth factor receptor (EGFRt) and CD19-CAR into HSPCs as well (205). These EGFRt-CD19-CAR modified cells can be removed by anti-CD19 antibody (cetuximab) treatment via ADCC. However, this ablation method is not as effective as the HSVsr39TK-ganciclovir system.

#### 4.1.3 Challenges & solutions

There are concerns about the impact of CAR transduction into stem cells like HSPCs. Zhen et al. observed that CD4-CAR substitutes endogenous CD3 expression and TCR recombination during CD4-CAR HSPC-derived T cell differentiation. T cells with high CD4-CAR expression showed reduced CD3/TCR levels (202). This suggests that T cells generated from CAR HPSCs most likely lose their ability to combat various pathogens and cancers. A solution might be the concurrent transplantation of both CAR-integrated and unmodified HSPCs. However, co-injecting multiple types of CAR T cells may lead to growth competition (206). It remains unclear if such competition will arise among T cells derived from different HSPC variants. Moreover, the CD28 costimulatory domain in CAR seemingly augments the tendency of CD19-CAR HSPCs to differentiate into NK cells. It is crucial to determine if CAR-modified HSPCs can maintain a balanced developmental and differentiation pathway, and the design of CAR constructs should be optimized based on the target cells and treatment goals. Furthermore, the risk of tumorigenesis remains for gene-modified HSPCs, especially when suicide genes/strategies failed to ablate all CAR cells. Extended *in vivo* examinations are required to further assess the safety of CAR HSPCs (**Table 1**).

### 4.2 CAR exosomes

#### 4.2.1 Properties and advantages

Exosomes, which are extracellular vesicles spanning 40-160 nm in diameter, are ubiquitously secreted by a vast majority of eukaryotic cells. They are of endosomal origin and formed by the invagination of both plasma and endosomal membranes. Characterized by their profound heterogeneity, exosomes contain a variety of membrane-associated protein complexes from their progenitor cells. This makes them crucial in facilitating intercellular communications (207). Studies have elucidated that exosomes secreted by T cells mediate the interaction between cytotoxic T cells and their target cells. These T cell-released exosomes carry TCR, CD8, and cytotoxic molecules, such as perforin and granzymes, indicating their potential capability to killing cells in an antigen-specific manner (208–210). Given this background, it is logical that exosomes from CAR T cells have unique cytotoxic properties as well. Indeed, CAR T cell-derived exosomes express CAR and have high levels of cytotoxic molecules (211). Therefore, CAR exosomes can target and kill tumor cells in a CAR-specific manner.

CAR exosomes, as cell-independent CAR derivatives, present several compelling advantages. Foremost, exosomes can easily penetrate deep into tissues and cross various physiological barriers (212). This suggests that CAR exosomes may have enhanced tumor infiltration ability towards solid tumors. Additionally, CAR exosomes provide an off-the-shelf therapeutic option for CAR treatments, given their cell-free nature. Moreover, the cytotoxic potential of CAR exosomes is independent of T cells, making them resistant to the suppressive effects of the TME. In addition, CAR exosomes tend to be safer than CAR T cells. This safety applies both during production, where there is no risk of tumorigenesis (due to its cell-free nature), and after administration. Lastly, exosomes and extracellular vesicles are being recognized as promising drug delivery methods (213). With their tumor-tropic ability, CAR exosomes could serve as an effective platform for precise drug delivery.

#### 4.2.2 Current study

Fu et al. developed exosomes with EGFR- and HER2-CARs from CAR-modified T cells (211). After antigen stimulation, these exosomes showed increased CAR expressions. Instead of undergoing uptake by target cells, these CAR exosomes selectively and directly lyse antigen-positive cells through granzyme B and perforin, displaying strong antitumor effects *in vivo*. Notably, these CAR exosomes did not express PD-1, making them resistant to PD-L1-mediated inhibition. In mouse models, the administration of these CAR exosomes resulted in no significant toxicities or CRS. Similarly, Yang et al. showed that mesothelin-CAR exosomes effectively suppressed tumor growth *in vivo* without evident toxicity (214). This effect comes from the direct killing of tumor cells by perforin and granzyme B on the CAR exosomes. Another study revealed that CD19-CAR exosomes induced contact-dependent cytotoxicity against CD19^+^ leukemia cell lines *in vitro* by upregulating pro-apoptotic genes (215). Interesting, this study noted that both CD19^+^ tumor cells and CD19^-^ control cells uptake CD19-CAR exosomes. This differs from Fu’s observation that target cells do not uptake CAR exosomes, However, only the uptake by CD19^+^ tumor cells led to a cytotoxic effect. It is postulated that cytotoxic effects occur only when CAR exosome entry is mediated by CD19 binding.

CAR exosomes have also been used to facilitate drug delivery. Zhu et al. developed a hybrid nanovesicle named Lip-CExo@PTX using exosomes from mesothelin- and PD-L1-bispecific CAR T cells. These exosomes were fused with lung-targeted liposomes and loaded with paclitaxel (PTX) (216). After intravenous administration into mice CT-26 metastatic lung cancer model, Lip-CExo@PTXs accumulated in the lung. There, they precisely released PTX and cytotoxic molecules towards mesothelin^+^ tumors, leading to sequential targeted delivery. The anti-PD-L1 ability of Lip-CExo@PTX further prevent immune suppression. Besides intravenous administration, the authors also developed PTX-loaded CAR exosomes (PTX@CAR-Exos), which can be administered via inhalation (217). In PTX@CAR-Exos, PTX is encapsulated into mesothelin-directed CAR exosomes. In orthotopic lung cancer mouse models, inhaled PTX@CAR-Exos accumulated within tumors and exert antitumor activity. Moreover, PTX@CAR-Exos also remodeled the immunosuppressive TME.

Besides CAR exosomes derived from CAR T cells, a system (called ExoCAR/T7@Micelle) has been developed. This system uses exosomes from CAR NK cells for drug delivery (218). In this system, CAR exosomes are obtained from HER2-CAR NK cells. Similar to exosomes from CAR T cells, these NK cell-derived exosomes also express HER2-CAR and show high affinity towards HER2^+^ breast cancer cells. Then T7 peptide is inserted into the exosomes for the binding with the transferrin receptor (TfR) on cerebral vascular endothelial cells. This process helps the exosomes cross the blood-brain barrier. The CAR exosomes are also loaded with a ROS-responsive nanobomb (mPEG-TK-Ce6@RSL3). This nanobomb is triggered in tumors with high levels of ROS [ROS is further amplified by a photodynamic therapy (PDT)-based strategy]. Both the released RSL3 and ROS can induce ferroptosis, exerting toxicity towards HER2^+^ tumor cells. In mice with orthotopic HER2^+^ breast cancer brain metastasis, ExoCAR/T7@Micelle exhibited enhanced antitumor activity. However, the study only focused on using NK cell-derived CAR exosomes for drug delivery. It did not investigate whether the NK cell-derived CAR exosomes have cytotoxic activities of their own.

#### 4.2.3 Challenges & solutions

While the aforementioned studies imply that the cytotoxicity of CAR exosomes relies on granzyme B and perforin, the precise mechanism remains unclear. Moreover, there is debate over whether target tumor cells uptake CAR exosomes. While Fu et al. demonstrated that CAR exosomes directly act on target cells without being taken up (211), other studies have reported that CAR exosomes are engulfed by target cells before releasing their cargo (215, 218). This uncertainty complicates our understanding of CAR exosomes’ activity and potential toxicity. For instance, T cell-derived exosomes carry TCRs from their progenitor cells, it is possible that CAR T cell-derived exosomes might also display endogenous TCRs. Consequently, we cannot ignore the potential alloreactivity of CAR exosomes. A deeper understanding of signal transduction within exosomes might clarify whether TCRs on CAR exosomes trigger downstream signaling pathways and the risk of GVHD.

Determining the optimal dosage of CAR exosomes for desired antitumor effects poses another significant challenge, especially since CAR exosomes do not proliferate. Given the inherent differences between CAR exosomes and CAR T cells, their cytotoxic effects are not directly comparable. Fu et al. found that CAR exosomes and CAR T cells exhibit similar *in vitro* cytotoxicity when they express comparable levels of CAR proteins, as measured by ELISA (211). However, it is unclear if this similarity extends to *in vivo* antitumor activity. Furthermore, the non-proliferative nature of CAR exosomes leads to uncertainty about their sustained *in vivo* activity, suggesting that multiple administrations might be necessary (**Table 1**).

## 5 Conclusions and future perspectives

While conventional CAR T cell therapy represents a pivotal advancement in cancer treatment, its clinical application is mainly limited to certain types of cancers. There are considerable challenges in improving the efficacy of CAR T cell therapy. These challenges also restrict its application to cancers beyond B cell-driven malignancies. Consequently, numerous studies have explored the use of CAR in different immune cells to overcome these challenges. Each cell type reviewed here have unique advantages that can potentially address the limitations of traditional CAR T cell therapy. A key feature of these cells is their ability to retain innate functions after CAR transduction. This enables them to exhibit CAR-independent antitumor activities alongside CAR-specific cytotoxicity. Another common feature is their MHC-independent activity, which reduces the risk of GVHD and pave ways for off-the-shelf CAR products. It is important to note that these CAR cells are reported safer than traditional CAR T cells. Yet, thorough *in vivo* studies are needed for a definitive safety assessment. While these CAR cells are theorized to have better tumor infiltration capabilities, verifying their actual accumulation within solid tumors is crucial. Lastly, many of the discussed CAR cells may modulate the immunosuppressive TME, but their effectiveness in the complex TME still needs to be proven (**Table 1**).

As mentioned earlier, a significant advantage of CAR products is their potential for off-the-shelf application. In addition to the novel CAR products discussed, there have been efforts to create allogeneic CAR T cells. The main strategy involves deleting or downregulating the TCR complex on CAR T cells to prevent GVHD (219–225). For instance, Hu et al. genetically removed HLA class II expression and the TCR/CD3 complex from CAR T cells to avoid T cell-mediated alloreactivity. To protect these modified CAR T cells from being attacked by host NK cells, they added the extracellular and transmembrane domains of E-cadherin to the CD28 intracellular domain. This addition creates an inhibitory receptor for NK cells (219). Comparing allogeneic CAR T cells with CAR cells from other sources is challenging due to differences in CAR design and the targeted antigens or diseases. Generally, clinical trials have shown that patients receiving allogeneic CAR T cells either experienced no GVHD (219, 224, 225) or only mild GVHD (mostly at skin or gastrointestinal system) (221, 223, 225), similar to those aforementioned CAR cells. It should be noted that the lack of alloreactivity may lead to poor *in vivo* expansion and reduced persistence, as previously discussed. To address this, some studies have deleted CD52 in allogeneic CAR T cells to provide a survival advantage over host CD52^+^ immune cells. This advantage comes into play upon the administration of anti-CD52 antibodies (221, 223, 226). Despite this, allogeneic CAR T cells still failed to expand in some patients (219, 221). Additionally, using anti-CD52 antibodies for lymphodepletion raises the risk of CMV reactivation (226). In contrast, the natural properties of other CAR cells might support their *in vivo* proliferation. For instance, the expansion of CAR γδ T cells can be promoted with amino bisphosphonates (82–84). However, the clinical application of this approach needs further exploration. Furthermore, gene deletion might diminish the effectiveness of CAR T cells (219, 220). There are also safety concerns associated with CRISPR-based gene editing (227). In comparison, CAR cells from other T cell subtypes don’t need as much genetic alteration. Thus, they maintain their antitumor activity and sidestep potential safety issues.

Poor persistence is a common issue for many new CAR products. However, it is not always detrimental, as faster clearance makes it easier to manage adverse effects. The major challenge is to balance potency and safety. While CD19-CAR T cells have prolonged persistence and can establish memory in patients, patients may also suffer from lifelong B cell aplasia (228, 229). Moreover, many of these new CAR cells can modulate the TME. Combining different CAR products can improve therapeutic efficacy or extend persistence (like the aforementioned synergistic effect of CAR macrophages and CAR T cells). For instance, since Th1 cells can convert TAMs to an M1 phenotype (17), and M1 macrophages can promote Th1 polarization (230), co-administering CAR macrophages with CAR NKT cells could be effective. M1-like CAR macrophages could enhance the activity and proliferation of Th1-like CAR NKT cells. In turn, Th1-like CAR NKT cells could help CAR macrophages retain their M1-like phenotype in the complex TME. This strategy can also be used by combining CAR cells derived from APCs like macrophages and γδ T cells with those from cytotoxic cells like T cells, NKT cells, and MAIT cells. Different CAR cells can amplify each other’s activity, fostering a sustained anti-tumor immune environment.

In the realm of CAR T cell therapy, concerted efforts have been directed towards improving its efficacy. These strategies could also benefit other CAR products, potentially leading to breakthroughs. To enhance persistence, IL-2 and IL-15 are often used. Similar approaches have been applied to CAR NKT cells, CAR γδ T cells, and CAR NK cells. Moreover, cytokines like IL-2, IL-7, and IL-15 can drive the expansion of CAR T cells (44). Using these cytokines in other CAR cells might boost their expansion in the absence of alloreactivity. Additionally, incorporating various interleukins can make CAR T cells more resilient to the immunosuppressive TME (44). This resilience could help CAR macrophages, neutrophils, and NKT cells maintain their anti-tumor phenotypes within the TME. Another challenge is that novel CAR cells tend to become exhausted. There have been many studies aimed at enhancing the fitness and stemness of CAR T cells, which may be helpful to other CAR cells as well. For example, designing CAR constructs carefully can minimize CAR T cell activation, reducing the risk of rapid exhaustion (3, 44). Adjusting CAR T cell metabolism and boosting mitochondrial functions can also extend their activity (231, 232). Although these new CAR cells are considered safe, concerns about potential on-target, off-tumor toxicity remain, such as CAR MAIT cells. The activity and potential toxicity of CAR T cells can be controlled through various logic-gating systems (233). Similar strategies could be adapted for CAR products from different cell sources. Combining these innovations in cellular immunotherapy with the unique features of these novel CAR cells, we look forward to future milestones in CAR cell therapy.

## Author contributions

JWH: Conceptualization, Investigation, Writing – original draft, Writing – review & editing. QY: Writing – review & editing. WW: Funding acquisition, Supervision, Writing – review & editing. JH: Conceptualization, Funding acquisition, Supervision, Writing – review & editing.
